# Exploring the Effectiveness of Self-Management Interventions in Type 2 Diabetes: A Systematic Review and Network Meta-Analysis

**DOI:** 10.3390/healthcare12010027

**Published:** 2023-12-22

**Authors:** Sofia Tsokani, Georgios Seitidis, Christos Christogiannis, Katerina-Maria Kontouli, Stavros Nikolakopoulos, Stella Zevgiti, Carola Orrego, Marta Ballester, Rosa Suñol, Monique Heijmans, Rune Poortvliet, Marieke van der Gaag, Pablo Alonso-Coello, Carlos Canelo-Aybar, Jessica Beltran, Ana I. González-González, Gimon de Graaf, Areti-Angeliki Veroniki, Dimitrios Mavridis

**Affiliations:** 1 Department of Primary Education, University of Ioannina, 451 10 Ioannina, Greece; 2 Methods Support Unit, Cochrane CET, London SW1Y 4QX, UK; 3 Department of Psychology, University of Ioannina, 451 10 Ioannina, Greece; 4 Avedis Donabedian Research Institute (FAD), 08037 Barcelona, Spain; 5 Netherlands Institute of Health Services Research, 3513 CR Utrecht, The Netherlands; 6 Iberoamerican Cochrane Centre, Biomedical Research Institute Sant Pau (IIB Sant Pau), 08025 Barcelona, Spain; 7 CIBER of Epidemiology and Public Health, CIBERESP, 28029 Madrid, Spain; 8 Institute for Medical Technology Assessment, Erasmus University, 3062 PA Rotterdam, The Netherlands; 9 Knowledge Translation Program, Li Ka Shing Knowledge Institute, St. Michael’s Hospital, Toronto, ON M5B 1T8, Canada; 10 Institute for Health Policy, Management, and Evaluation, University of Toronto, Toronto, ON M5T 3M6, Canada

**Keywords:** network meta-analysis, self-management interventions, component network meta-analysis, type 2 diabetes

## Abstract

Background: Chronic diseases are a leading cause of global morbidity and mortality. In response to this challenge, self-management interventions (SMIs) have emerged as an essential tool in improving patient outcomes. However, the diverse and complex nature of SMIs pose significant challenges in measuring their effectiveness. This work aims to investigate the comparative effectiveness of SMIs on Type 2 diabetes mellitus (T2DM) outcomes. Methods: A rigorous analytical framework was employed to assess the relative effectiveness of different SMIs, encompassing both pairwise and network meta-analysis (NMA), as well as component network meta-analysis (CNMA). Various outcomes were considered, including glycated hemoglobin (HbA1c) control, body mass index (BMI) reduction and low-density lipoprotein (LDL) cholesterol. Visualization tools were also utilized to enhance the interpretation of results. Results: SMIs were found promising in improving clinical outcomes and patient-reported measures. However, considerable heterogeneity and inconsistency across studies challenged the validity of NMA results. CNMA along with various visualization tools offered insights into the contributions of individual SMI components, highlighting the complexity of these interventions. Discussion/Conclusions: SMIs represent a valuable approach to managing chronic conditions, but their effectiveness is context-dependent. Further research is needed to elucidate the contextual factors influencing SMI outcomes. This work contributes to a comprehensive understanding of SMIs’ role in T2DM management, aiming to aid decision-makers, clinicians, and patients in selecting tailored interventions.

## 1. Introduction

Chronic diseases refer to long-term health conditions that involve ongoing medical attention, and the most common include diabetes mellitus, cancer, heart failure, arthritis, and chronic obstructive pulmonary disease (COPD), among others [1]. Worldwide, chronic diseases are the leading cause of death for all ages, genders, and ethnicities, and, consequently, strain is being placed on the healthcare system and society as a whole [2]. In Europe, over 80% of elder people (aged over 65) suffer from chronic conditions, contributing to 77% of the total disease burden gauged in disability-adjusted life years (DALYs) [3]. Diabetes is one of the fastest growing global health chronic conditions of the 21st century. According to the International Diabetes Federation (IDF), 537 million people suffer from diabetes worldwide, with this number expected to rise to 643 million by 2030 and to 783 million by 2045 [4].

Empowering individuals to manage their health has become a pivotal strategy in addressing the challenges posed by chronic diseases [5]. This signals a shift from the traditional model, where patients are seen as passive recipients of care, marking the beginning of a more balanced and collaborative clinician–patient relationship [6].

Self-management interventions (SMIs) are supportive interventions systematically provided by healthcare professionals or other patients with the purpose of strengthening patients’ confidence and actively engaging them in the management of their health conditions to promote behavioral change [7]. They typically comprise various components, such as the mode of delivery and the type of recipient. These components may interact over time as participants transition between intervention processes and daily life, posing challenges in accurately measuring their effectiveness. SMIs may range from the provision of condition-specific information via a website or a leaflet to extensive self-management support programs such as the Expert Patients Programme [8].

Although SMIs may differ in content across and within conditions, they share a common goal, which is to improve the patients’ ability to effectively cope with the daily challenges imposed by their chronic condition. In essence, SMIs aim to develop self-efficacy so that people can actively participate in the continuous management of their medical condition, build resilience, and enhance their general sense of wellbeing. Key self-management behavioral skills include targeted abilities such as problem-solving, the efficient utilization of resources, and the improvement of patient/healthcare provider communication.

The COMPAR-EU project, funded by the European Union, is a multidisciplinary initiative aimed at identifying the most promising SMIs, in terms of both effectiveness and cost-effectiveness, for adults facing four high-priority chronic conditions: type 2 diabetes mellitus (T2DM), obesity, heart failure, and COPD [9]. Also, it endeavors to bridge the gap between existing knowledge and the practical implementation of SMIs. Despite a general trend of positive results, the rapidly expanding number of SMIs, the huge diversity among them, and the variety of reported effectiveness results makes it extremely complicated to decide which interventions are the most effective and/or cost-effective for different purposes and in different contexts [10].

Within the COMPAR-EU context, we acknowledge that SMIs play an important role in addressing the growing burden of chronic diseases. To date, evidence on the effectiveness of SMIs has been mainly based on results from pairwise meta-analyses of randomized controlled trials (RCTs). Pairwise meta-analysis, however, does not allow for simultaneously comparing multiple SMIs and does not provide any insight regarding the effectiveness of the SMIs’ individual components. We posit that the application of network meta-analysis offers a unique opportunity to move beyond the limitations of pairwise analyses.

To assess the comparative effectiveness of SMIs for patient important outcomes in adults with T2DM, we conducted a network meta-analysis and a component network meta-analysis [11,12]. Through this approach, we aimed to uncover those SMIs and their underlying components that exhibit promising results, contributing to a clearer understanding of how these interventions can be tailored to meet the diverse needs of patients with T2DM across various contexts and purposes.

## 2. Methods

### 2.1. Design

The protocol for this systematic review and NMA has been previously registered in Open Science Framework (OSF; https://osf.io/65zgr) [13]. Briefly, we conducted a systematic review using standard NMA and component NMA [12,14,15,16]. We followed the Preferred Reporting Items for Systematic Reviews and Meta-Analyses extension statement for network meta-analysis (PRISMA-NMA) guidelines to ensure adequate reporting (Supplementary Material S1) [17].

### 2.2. Search Strategy

To identify relevant RCTs, we conducted a search on the databases of a previous European project, named PRO-STEP, that located RCTs on SMIs for diabetes, published from 2000 up to 2015 [18]. We updated the database by searching in PubMed, CINAHL, Embase, Cochrane, and PsycINFO. There was no restriction regarding country in the search strategy, whereas the primary languages were English and Spanish, as these were the languages of the COMPAR-EU consortium. We included RCTs that compared SMIs in adults with T2DM and were published in English or Spanish. The search targeted RCTs published between 2015 and 2018, to enhance the findings of PRO-STEP’s systematic review. The potential inclusion of earlier years was evaluated in cases where PRO-STEP did not cover those previous years sufficiently. Quasi-randomized studies were excluded.

SMIs for adults (>18 years) with T2DM were included, with the type of population being patients or caregivers. If a mixed population of diabetes was included, i.e., patients with Type 1 and Type 2 diabetes mellitus, the study was included if at least 80% of the participants have T2DM or when results are given separately for each diabetes type. Interventions targeting health professionals only were excluded. The search strategy and inclusion criteria for the RCTs assessing SMI for T2DM in adults are summarized in Supplementary Material S2.

### 2.3. Study Selection and Data Extraction

Two reviewers independently screened studies for inclusion. Data were extracted by one reviewer and verified by a second one. A third reviewer was consulted to resolve any disagreements. From the included studies, we extracted and collected data regarding patient characteristics, disease characteristics and comorbidities, intervention characteristics, outcomes, and results, as well as information on study design and risk of bias.

### 2.4. Risk of Bias Assessment

One reviewer assessed each included study for risk of bias utilizing the Cochrane risk-of-bias tool. [19]. A second reviewer validated the assessments. We resolved any disagreements with discussion and the involvement of a third reviewer. For each study, the risk of bias was rated as low risk, high risk, or unclear across the five tool domains. In cases where relevant information regarding the risk of bias domains was missing, we reached out to the study authors to request clarifications or additional details.

### 2.5. Statistical Analysis

We synthesized the evidence using three different types of analysis: single-effect analysis, network meta-analysis (NMA), and component network meta-analysis (CNMA). The single-effect analysis aims to explore whether SMIs work in general. We observed significant variability within the control group, which prompted us to distinguish between two categories: “usual care” (UC) and “usual care plus” (UCP), with the latter representing a more active approach to usual care. Most studies compared an SMI to UC or UCP, and we were able to estimate an effect for SMIs irrespective of the components the SMI consisted of. NMA is an established methodology for comparing different SMIs [11,12]. We expected that networks would be sparse, with most SMIs being compared in a couple of studies. In such cases, NMA results may be confounded with study characteristics since NMA effect estimates are mainly informed by a few studies and the transitivity assumption is doubtful. We conducted an additive CNMA so that each component effect is informed by all studies including that specific component [14,16]. The additivity assumption is also a strong one, and it is likely that components interact synergistically or antagonistically with each other [20]. We also employed a series of visualization tools to NMA results to explore how components are associated with the SMIs’ effectiveness [21].

All the outcomes examined were continuous. Therefore, in our analyses, we employed the mean difference (MD) as effect size or the standardized mean difference (SMD), if the included studies reported results on multiple different scales, accompanied by the corresponding 95% confidence intervals (CI).

#### 2.5.1. Single-Effect Analysis (SEA)

The aim of the single-effect analysis was to explore if SMIs work in general. Within each outcome, we selected all interventions directly comparing an SMI to UC/UCP. We did not differentiate between different SMIs resulting in a single comparison (SMI vs. UC/UCP). We performed a standard pairwise meta-analysis of all SMIs versus UC/UCP using both fixed and random-effects models, to explore the effect of SMIs globally. We also included the heterogeneity estimator (τ^2^-restricted maximum likelihood estimator). The Hartung–Knapp–Sidik–Jonkman (HKSJ) method was applied for calculating 95% confidence intervals [22,23]. Prediction intervals (PI) were used to examine how heterogeneity affects the summary estimate and the resilience of the results to future trials [24]. Utilizing funnel plots and Egger’s test, we assessed small-study effects both graphically and statistically [25].

We performed meta-regression analyses, using baseline risk (severity) and gender (percentage of females) as covariates, and a subgroup analysis to explore the association between the SMIs’ effectiveness and intensity, which was classified in two levels: high and low intensity.

#### 2.5.2. Network Meta-Analysis (NMA)

NMA is a well-established statistical method that synthesizes direct and indirect evidence to produce the relative effect estimates between any pair of interventions within a network of interventions [11,26]. For each outcome, we conducted a random-effects network meta-analysis (NMA) within the largest connected subnetwork. The analysis was performed using a random-effects NMA model within a frequentist framework, utilizing methods derived from graph theory [26,27]. All effect sizes were given along with the corresponding 95% confidence intervals (CI). Prediction intervals (PI) were also estimated.

A fundamental assumption of NMA is transitivity, according to which all the studies included in direct comparisons should be sufficiently similar [12]. The statistical analogue of transitivity is consistency, which implies the (statistical) agreement between the direct and indirect comparisons. We used the “design-by-treatment” interaction model and the node-splitting approach to check for inconsistency in the entire network (globally) and in each individual loop (locally), respectively [28]. We also provide P-scores (the probability for each intervention to be better than all competing interventions) for all interventions per network [29].

#### 2.5.3. Component Network Meta-Analysis

SMIs are multicomponent interventions and consist of multiple, possibly interacting components. We applied CNMA to explore individual components’ effects. While standard NMA treats each combination of components observed in data as a distinct intervention, CNMA estimates the effect of each component and subsequently the effect of each SMI by summing the effects of the components constituting this SMI (additivity assumption) [14,15,20]. CNMA allows for estimating the effects of individual components and identifying the most effective ones. The summary absolute effects of all components were obtained along with their corresponding 95% CIs.

#### 2.5.4. Visual Inspection of NMA Results Using a Series of Visualization Tools

We employed a series of visualization tools to explore the components’ behavior according to standard NMA results for each outcome [21]. The graphs showed how the components acted and which ones were most effective. In addition to providing valuable insight into the network’s geometry, this approach also provided insight into the effectiveness of each component. To gauge the components’ effectiveness, we used a range of suggested visualization tools including component descriptive analysis, leave-one-component (combination) out scatter plots, violin plots, density plots, and component heat plots. The component descriptive analysis examines the frequency of the components in the NMA model. The leave-one-component (combination) out scatter plot allowed us to investigate whether the inclusion or exclusion of a specific component (or combination) impacts on the intervention’s effectiveness. The violin plots offered a comprehensive view of their distributions, either individually or in groups. Density plots aided in comparing the relative effects of interventions including certain component(s) against those without the specific component(s). Additionally, based on the NMA estimates, component heat plots were created to explore the efficacy of component pairs of interventions. Finally, the rank-heat plot summarized the effectiveness of components across multiple outcomes, providing a visual representation of their hierarchy, based on the median of the intervention P-scores including the component of interest in the particular outcome. All tools are available in the viscomp R-package [30].

### 2.6. Certainty of Evidence Assessment

We rated the certainty of evidence obtained from pairwise meta-analysis and network meta-analysis using the GRADE approach guidance (Grading of Recommendations, Assessment, Development, and Evaluations). Additionally, we applied the CINeMA framework (Confidence in Network Meta-Analysis) to evaluate the confidence in the NMA findings by accounting for the network’s evidence flow and considering the impact of studies with high or unclear risk of bias within the network [31].

## 3. Results

### 3.1. Interventions

SMIs are multicomponent interventions which, according to our taxonomy and discussion within the COMPAR-EU consortium, consist of eleven different components, based on the taxonomy of SMIs for patients with long-term conditions suggested by Orrego et al. [32]. These are action-based behavioral change techniques (AB), education (E), emotional-based behavioral change techniques (EB), monitoring techniques (MT), shared decision making (SD), and social support (SS). We also considered whether the SMI was given by peers and/or lay person (P), remotely (R), and in groups (G). When component P was not present, we considered both peers and professionals, when component R was not present, we considered that the SMI was delivered face-to-face, and when component G was not present, we considered that the intervention was delivered both in groups and individually. There was much heterogeneity in the control group, and for this reason we differentiated between usual care (UC) and what we called usual care plus (UCP) with the latter being a more active UC [32].

A total of 665 studies were eligible for inclusion; the studies were conducted across 64 different countries and included 164,437 T2DM adults in total. Approximately one in five studies (21%) were carried out in Europe, while the remaining 79% were conducted outside of Europe. Most of these studies came from the United States (35%), followed at a distance by Iraq (7%), United Kingdom (6%), China (6%) and Korea (5%).

In the 665 studies analyzed, the number of intervention arms per study varied from two to five. Most studies (90%) featured two arms. A total of 530 studies (80%) compared a SMI to usual care, whereas 135 studies involved comparisons between one or more intervention arms (head-to-head). Usual care, as defined by the authors, typically involved regular visits and education. However, in some studies, it extended beyond information or education to include additional elements such as skills training or coaching, termed as “usual care plus”.

Twenty-one outcomes, organized in five broad categories, were included in total, as outlined in Table 1. We performed network meta-analysis for all outcomes; in the main manuscript, we report results on glycated hemoglobin (HbA1c), body mass index (BMI), and low-density lipoprotein (LDL) cholesterol outcomes. The flow chart can be found in Supplementary Material S2, whereas results from all analyses for the remaining outcomes are available in both Supplementary Materials S3–S10 and the COMPAR-EU platform (https://platform.self-management.eu/).

### 3.2. Risk of Bias of Included Studies

Most of the studies were at low risk of bias in the sequence generation of the random number for the allocation of participants. There was a lack of clarity in reporting the methods for allocation concealment, whereas a significant proportion of studies was judged as unclear. The primary methodological limitation of the included studies was the lack of blinding of the intervention concerning both participants and personnel. Only a few studies implemented a method to conceal the active group from other participants or care personnel or employed a “sham” intervention to minimize the impact of participants being aware of their allocated study arm. This limitation also impacted on the assessment of both subjective (such as quality of life, self-efficacy, knowledge, etc.) and objective outcomes that could be influenced by the assessor (such as BMI, waist size, etc.). We considered that objective outcome assessments from laboratory tests or clearly observed events (such as mortality or glycated hemoglobin) were not influenced by the absence of blinding. Assessing the risk of selective reporting proved challenging since only a limited number of studies provided their protocols before publishing the results. The risk of bias per outcome for each study is presented in Supplementary Material S9.

### 3.3. Single Effect (Pairwise Meta-Analysis) Results

In a pairwise meta-analysis of studies comparing SMIs with UC/UCP using a random-effects model, a mean difference of −0.39% [95% CI −0.45 to −0.34] was found for the HbA1c. For BMI, the summary mean difference was −0.28 kg/m^2^ [95% CI −0.42 to −0.15] and for LDL cholesterol it was −1.78 mg/dL [95% CI −3.02 to −0.53]. In all three outcomes, there was substantial between-study heterogeneity as measured using τ^2^ and quantified using the index I^2^ in Table 2. Overall, SMIs tend to reduce levels of HbA1c and BMI; yet, results are not conclusive, and this is reflected in the wide prediction intervals. The prediction intervals include the value of no effect (i.e., MD = 0); therefore, there is weak evidence to support the effectiveness of SMIs compared to UC/UCP for the studied outcomes in a potential future study. Similar patterns were observed for most outcomes, as presented in Supplementary Material S3. Regarding the rest of the outcomes, random-effects meta-analyses gave statistically significant results for all outcomes except for reducing high-density lipoprotein (HDL) cholesterol outcome. These results suggest that SMIs are effective compared to UC/UCP. However, heterogeneity was substantial in all analyses, ranging from 66% to 96%, and all prediction intervals crossed the line of no effect, suggesting that the results are not conclusive. Small-study effects were present in half of the outcomes assessed. The results of the pairwise meta-analysis under the fixed-effect model are available in Supplementary Material S3. The differences in estimates between fixed and random-effects models, deriving from the pairwise meta-analyses of SMIs vs. UC/UCP, suggest the presence of small-study effects. Egger’s test also suggests that there is evidence for small-study effects for all three outcomes described above (see also funnel plots in Supplementary Material S4).

We performed a subgroup analysis to explore if the effectiveness of SMIs is associated with intensity. Intensity (measured in hours) was not reported in all studies, and in multi-arm studies, there were cases where the different arms of SMIs were using different intensities. Such comparisons of SMIs vs. UC/UCP were treated as separate comparisons, ignoring the dependence between effects within the same trial. The subgroup analysis results show that for HbA1c, both the high and the low intensity of the intervention have a similar impact. However, in BMI and LDL cholesterol, high intensity may have a better effect on the outcome; yet, due to large heterogeneity and overlapping confidence intervals, the results are not conclusive (Table 3).

We performed a meta-regression using as a covariate the baseline risk, which was considered a proxy of severity of disease; the results showed that the baseline risk did not significantly impact on HbA1c (−0.01; 95% CI [−0.01 to 0.00]; I^2^ = 98%), and the same holds true for BMI (−0.01; 95% CI [−0.03 to 0.01]; I^2^ = 93%), and LDL cholesterol (−0.09 [−1.24 to 1.07]; I^2^ = 99%). Similarly, when conducting meta-regression with the percentage of females as a potential effect modifier, our findings suggested that gender did not have a significant impact on the outcomes (HbA1c (0.00; 95% CI [0.00 to 0.01]; I^2^ = 86%), BMI (0.03; 95% CI [0.01 to 0.05; I^2^ = 87%]), and LDL cholesterol (0.13 [−0.07 to 0.33]; I^2^ = 96%)). It is worth noting that for BMI results, while statistically significant, they lack practical significance as the effect estimates are close to zero. The subgroup and meta-regression analysis results for the rest of the outcomes are also provided in Supplementary Materials S6 and S7, respectively.

### 3.4. Network Meta-Analysis Results

**Figure 1:**
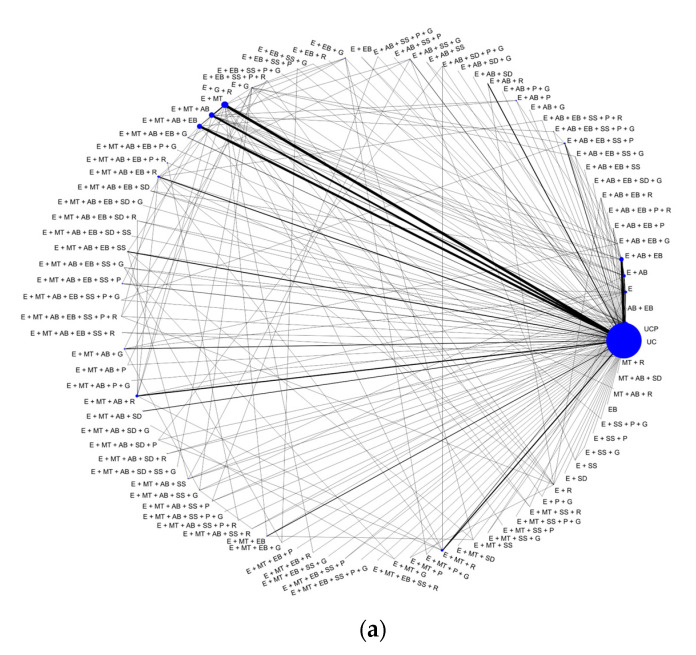

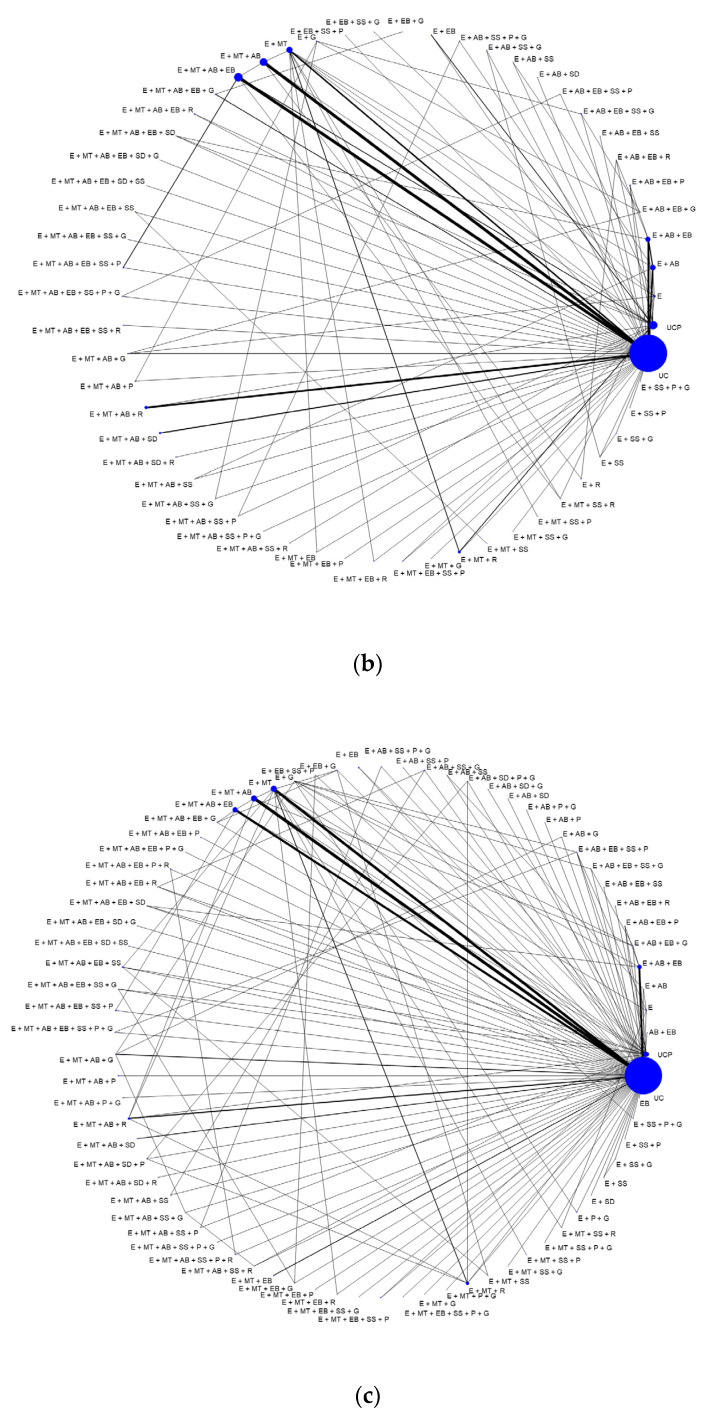
Network plots for (**a**) HbA1c, (**b**) BMI, and (**c**) LDL cholesterol.

Figure 1 shows the network plot for HbA1c, BMI, and LDL cholesterol. We see that networks are sparse, consisting of many nodes and mainly trials comparing an SMI to UC or UCP. This was a common theme encountered in all network plots for all outcomes. Most of the networks were originally disconnected, with a few trials comparing SMIs that are not included in the big network; we performed an NMA in the largest connected network in all outcomes.

The results for the most efficacious interventions, judged as those with a P-score larger than 0.80, are shown in Table 4. It should be noticed that the between-study variance (heterogeneity) in the three networks was quantified as high; using the design-by-treatment interaction model for inconsistency, a substantial variability across studies was observed for both outcomes.

For the HbA1c outcome (Figure 1a), the evidence network included 465 studies evaluating 97 multicomponent interventions. According to NMA results (Table 4), the most efficacious interventions were E + EB + SS + G (MD −1.42%; 95% CI [−2.02 to −0.82]), E + MT + EB (MD −0.78%; 95% CI [−1.00 to −0.57]), and E + SS + G (MD −0.69%; 95% CI [−1.04 to −0.35]). For BMI, 230 studies explored 76 interventions (Figure 1b) while the most efficacious interventions included E + AB + EB + SS (MD −1.88 kg/m^2^; 95% CI [−2.89 to −0.88]) and E + MT + P + G (MD −1.70 kg/m^2^; 95% CI [−3.03 to −0.37]). Regarding LDL cholesterol, 171 studies evaluating 58 multicomponent interventions were included in the corresponding network (Figure 1c). In terms of effectiveness for reducing LDL cholesterol, E + MT + SS + P (MD −35.10 mg/dL; 95% CI [−42.35 to −27.84]), E + MT + SS + R (MD −16.63 mg/dL; 95% CI [−21.72 to −11.54]), and E + AB + SD (MD −15.47 mg/dL; 95% CI [−25.71 to −5.23]) appeared to be among the most efficacious interventions.

Similar tables for the rest of the outcomes are given in Supplementary Material S5. A common theme that we observed in all outcomes is that NMA effects for most interventions were informed mainly by single studies comparing these interventions, and therefore there is a strong likelihood of confounding them with study characteristics.

### 3.5. CNMA Results

We conducted CNMA including studies disconnecting the networks. In contrast to NMA, which can be applied only to connected networks, CNMA is applicable to disconnected networks if the subnetworks share at least one common component [16,32]. Table 5 displays the CNMA results per component for the HbA1c, BMI, and LDL cholesterol outcomes.

The education component (E) seems to be effective for both HbA1c (MD −0.25%; 95% CI [−0.34 to −0.16]) and LDL cholesterol (MD −1.84 mg/dL; 95% CI [−3.46 to −0.23]). The between-study heterogeneity was high for all outcomes when applying the CNMA model. The component effects for the rest of the outcomes are shown in Supplementary Material S8. The education component (E) is statistically significant favoring SMIs in the following outcomes: adherence, diastolic blood pressure, dietary habits, foot care, HbA1c, HDL cholesterol, knowledge, LDL cholesterol, physical activity, psychological distress, and systolic blood pressure. Statistically significant component effects were found for components AB (care satisfaction, waist size, and weight), EB (psychological distress, self-efficacy, and weight), MT (HbA1c and systolic blood pressure), SD (adherence and glucose self-monitoring), and SS (knowledge, self-efficacy, and waist size).

### 3.6. Visual Inspection of NMA Effects

**Figure 2:**
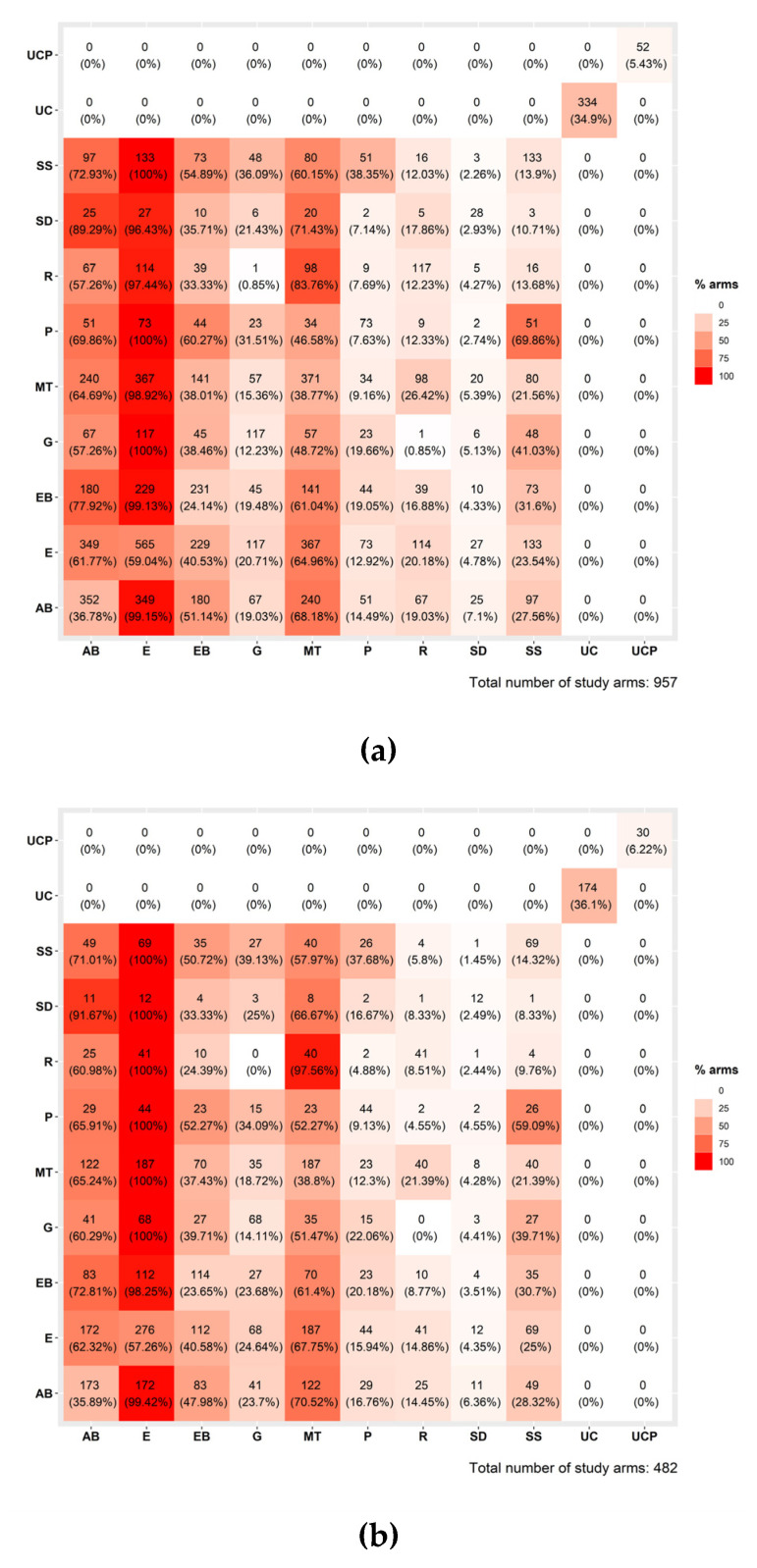

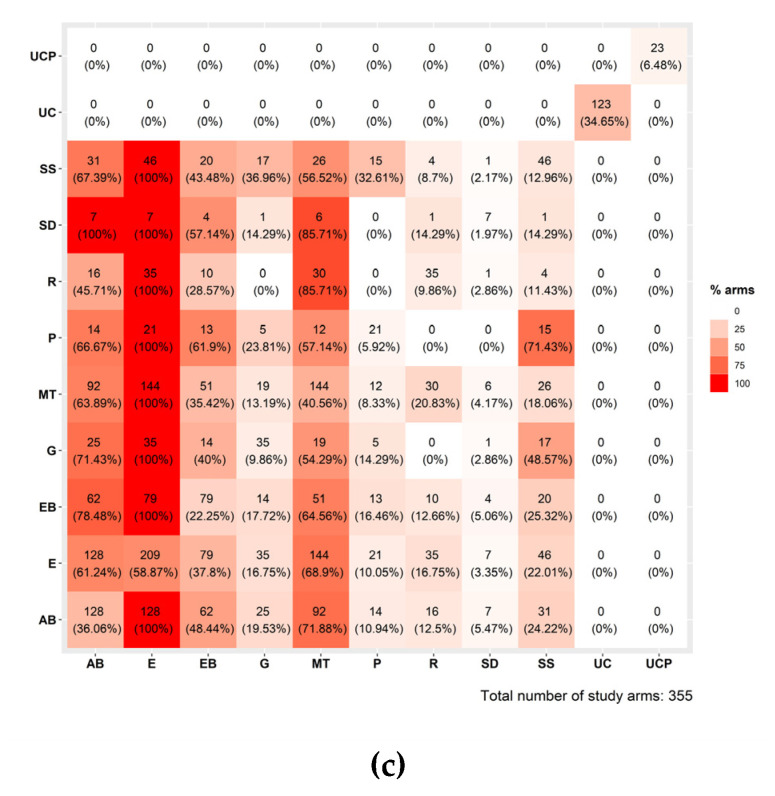
Component descriptive analysis for (**a**) HbA1c, (**b**) BMI, and (**c**) LDL cholesterol.

We used the viscomp R-package [21,30] to produce a series of plots to explore which components are more promising, based on the Z-values (or else standardized effects, i.e., TE/standard error of TE). Based on the component descriptive analyses depicted in Figure 2a–c, most of the interventions included the education (E) component, while the controls UC and UCP were not combined with any other components.

**Figure 3:**
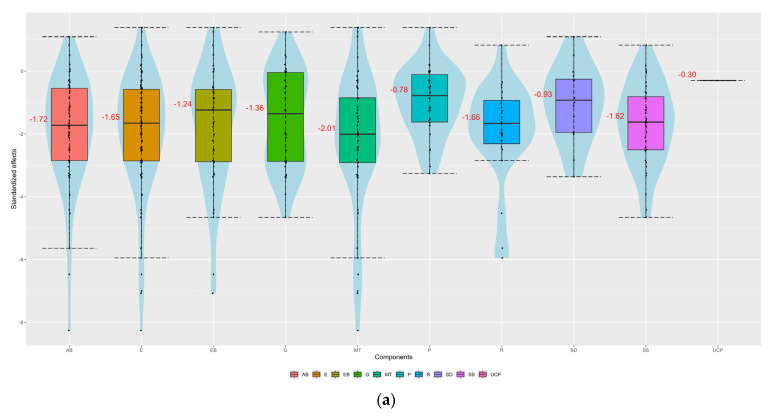

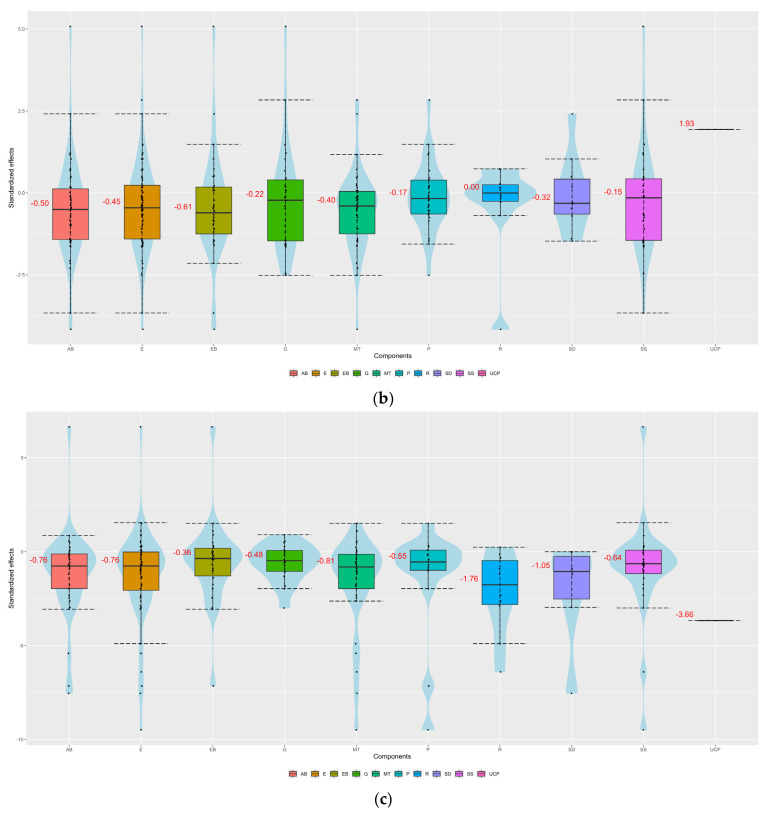
Violin plots for (**a**) HbA1c, (**b**) BMI, and (**c**) LDL cholesterol.

**Figure 4:**
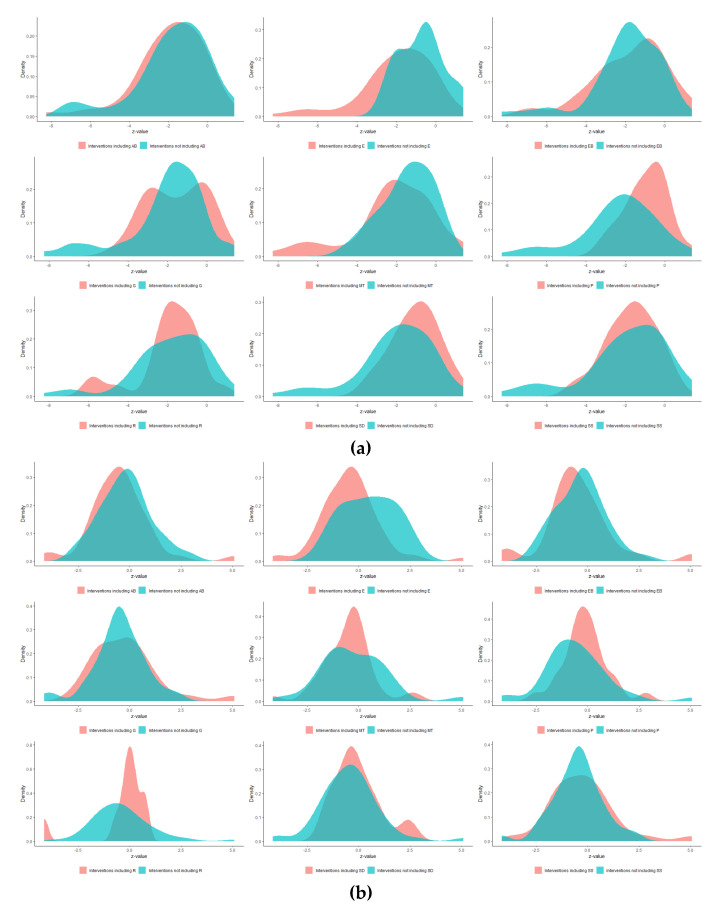

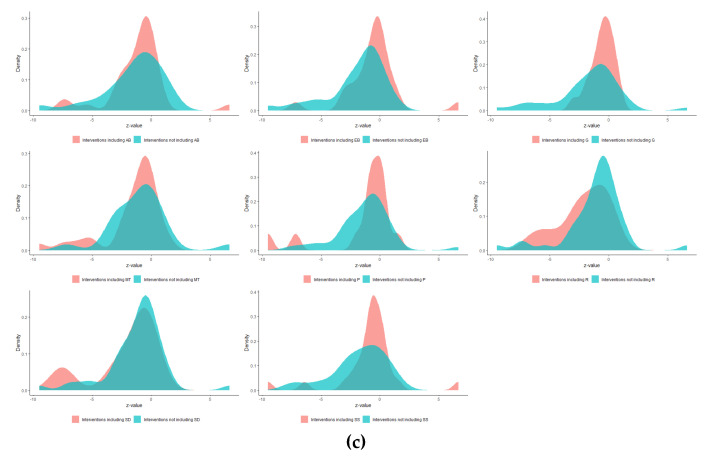
Density plots for (**a**) HbA1c, (**b**) BMI, and (**c**) LDL cholesterol.

**Figure 5:**
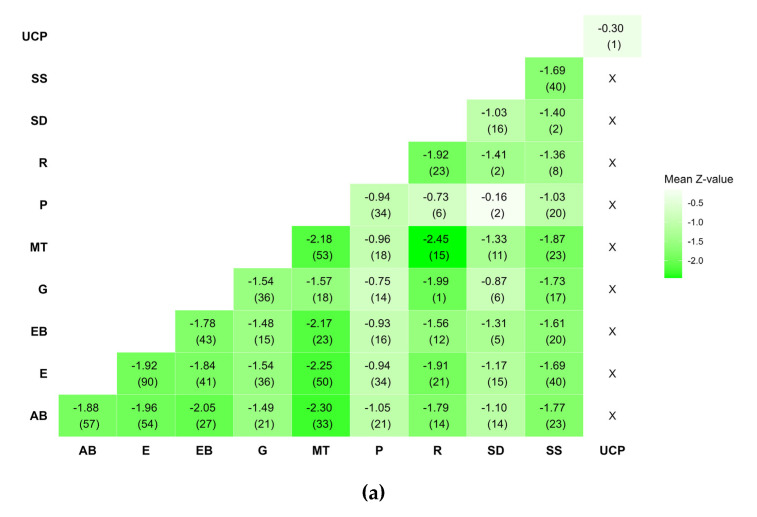

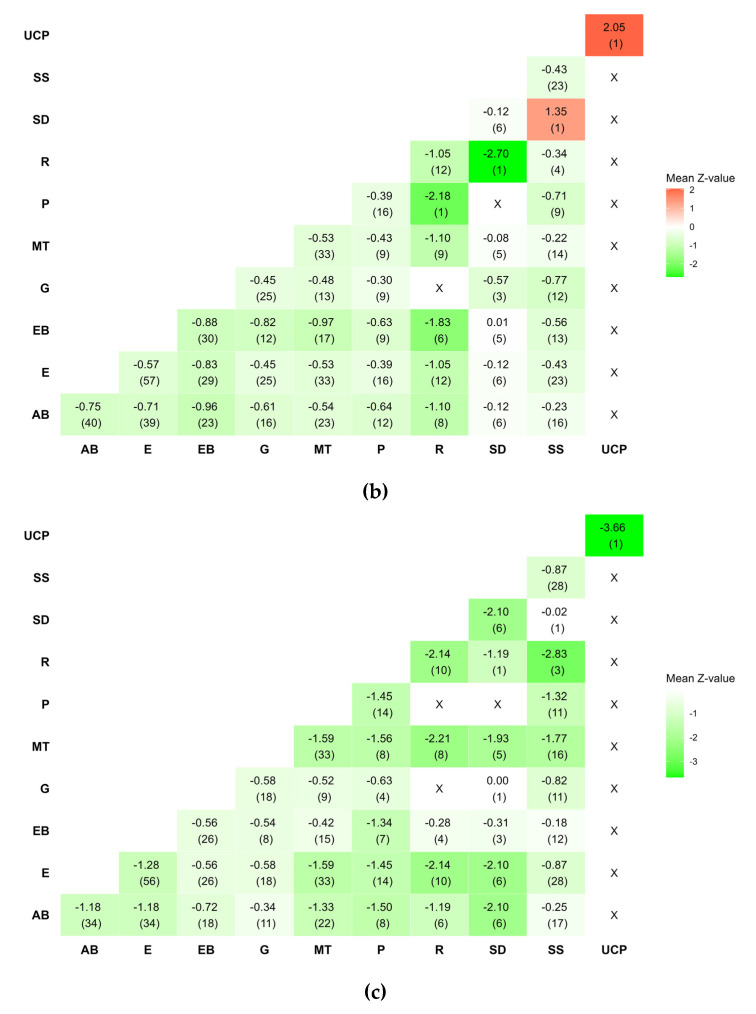
Component heat plots for (**a**) HbA1c, (**b**) BMI, and (**c**) LDL cholesterol.

Figure 3a–c show that all components (except UCP, which is a single intervention) had a similar positive impact on all three outcomes. In terms of reducing HbA1c and LDL cholesterol, components MT and SS, respectively, may perform slightly better. Regarding HbA1c, components SD and R led to an increased strength of statistical evidence (the interventions including them perform better than UC). Additionally, there was an increased strength of statistical evidence that the interventions not including component P are better than the interventions including it (Figure 4a). This was also the case for component MT which may perform better than UC when combined with most of the components, and especially with AΒ and R (Figure 5a).

**Figure 6:**
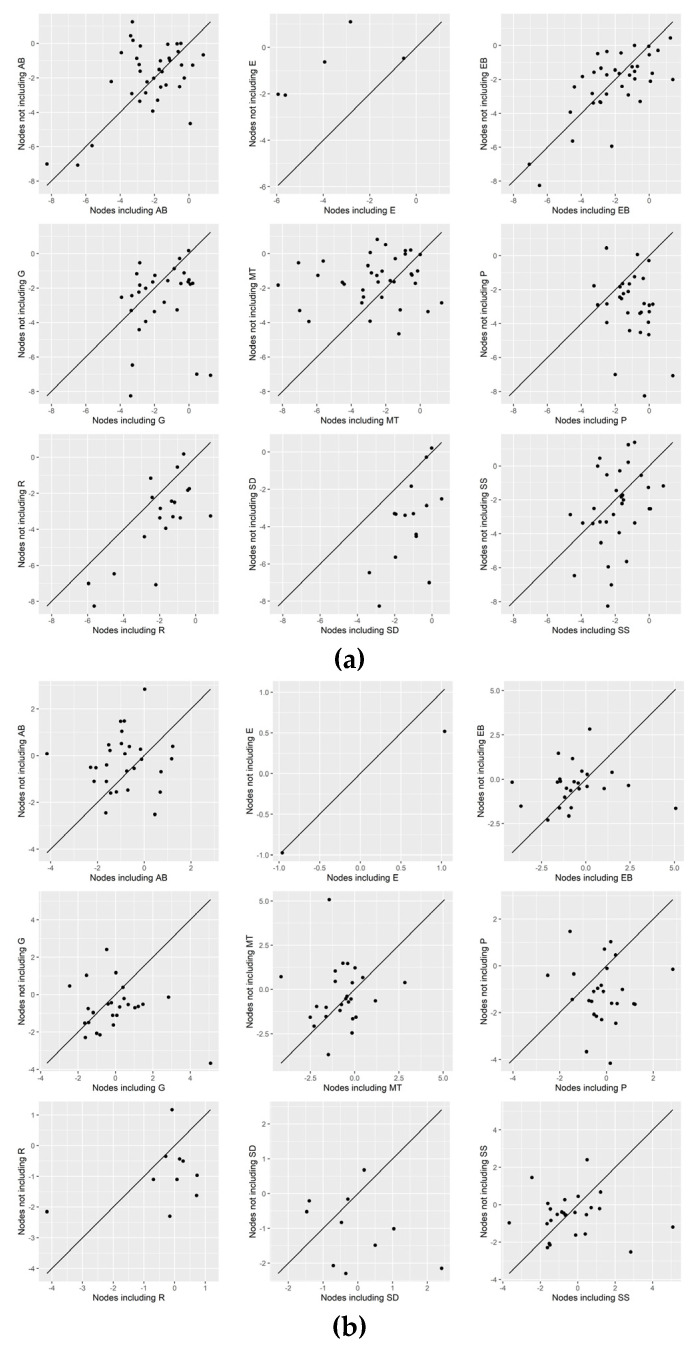

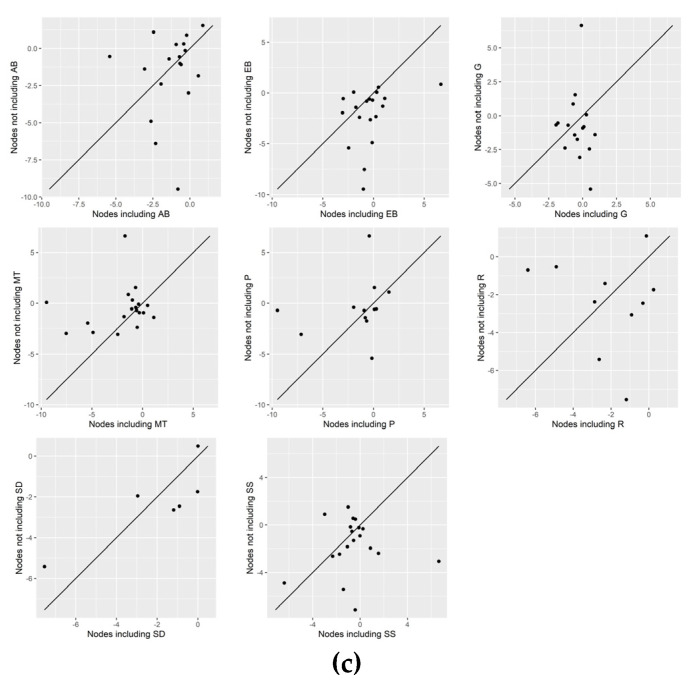
Leave-one-component-out scatter plots for (**a**) HbA1c, (**b**) BMI, and (**c**) LDL cholesterol.

In BMI, the less promising component seems to be SD, for which Figure 4b shows that the strength of statistical evidence is not drastically increased when combined with the rest of the components; in more detail, a rise in BMI Z-value was observed when combined with EB. By comparing standardized treatment effects that differ by a single component, only components R and P were found to increase the Z-values when included (Figure 6b). There is not much information on interventions that differ by component E because, as mentioned above, it was included in most interventions.

Concerning LDL cholesterol, most pairwise combinations of components are effective when included in interventions compared with UC; the combination of R and SS appears to be interesting because, compared to other combinations, it results in a greater reduction in the Z-values of LDL cholesterol.

**Figure 7:**
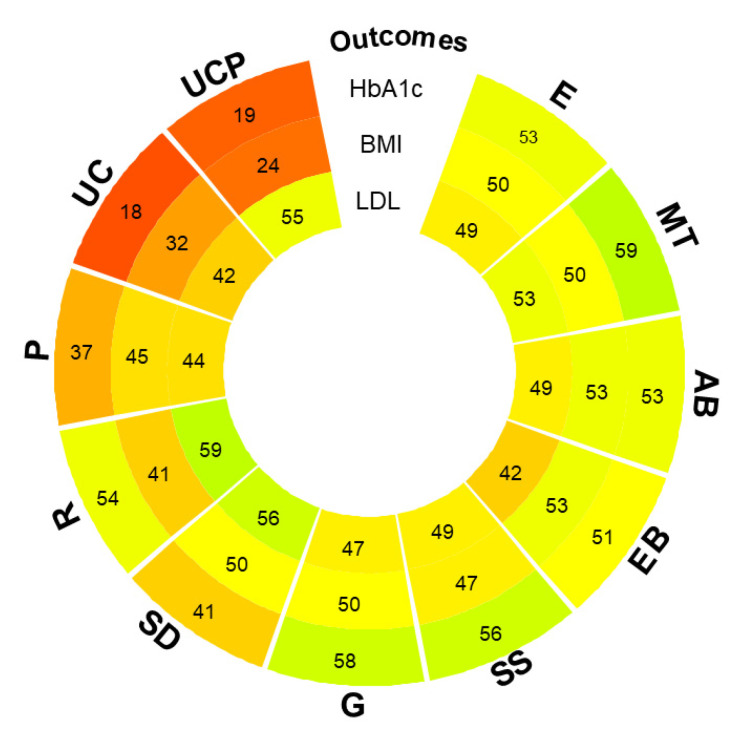
Rank heat plot for HbA1c, BMI, and LDL cholesterol.

Overall, a rank heat plot was used to rank interventions across all main outcomes and provide a summary of information and evidence; the median of P-scores of interventions including each component of interest for each outcome was used to rank interventions across all main outcomes. When included in interventions, MT and G appear to be more effective across all three outcomes (Figure 7).

### 3.7. Confidence in NMA Results

CINeMA analyses showed that we have major concerns for imprecision, within-study bias, heterogeneity, and inconsistency for most comparisons in all outcomes. CINeMA results for each outcome for comparisons of all interventions versus UC can be found in Supplementary Material S10. Results on GRADE assessment of outcomes can be found in the COMPAR-EU platform (https://platform.self-management.eu/).

## 4. Discussion

There is evidence to suggest that SMIs for chronic conditions can improve clinical outcomes, such as reduction of glycated hemoglobin (HbA1c) in patients with T2DM. SMIs have also been associated with the improvement of patient-reported outcomes such as quality of life, self-efficacy and related measures, and adherence measures. Also, SMIs have been related to positive results in terms of cost-effectiveness measured from an individual and societal perspective. SMI research has therefore blossomed over the past ten years, with over 26,100 articles published in PubMed. Furthermore, the European mobile Health (mHealth) Market is expanding fast. Since 2013, it has been growing at an annual rate of 54%, and a large proportion of this market is attributed to SMIs.

The meta-analysis results indicated that SMIs were effective when compared to UC/UCP. However, it is important to note that substantial heterogeneity was observed in all analyses. Networks of SMIs have certain characteristics present across all outcomes analyzed in this systematic review. These include many distinct SMIs, sparseness with most SMIs not directly compared to each other, many trials comparing an SMI to UC, substantial heterogeneity, and inconsistency. With multiple treatment comparisons, NMA is considered the standard approach to make inferences about the relative effectiveness of SMIs. The validity of NMA results rests on the plausibility of the assumptions made, and the fundamental NMA assumption of transitivity is challenged. This is also evident from the large inconsistency and heterogeneity observed in all networks for all outcomes. In all outcomes, we see that NMA results are confounded with study characteristics and do not reflect actual SMI effectiveness. This is something that we expected from the beginning, and to this end, we conducted a series of additional analyses. Though the single-effect analysis shows large heterogeneity, it seems that SMIs work for most of the outcomes (the exceptions are HDL and psychological distress). The large statistical heterogeneity may reflect the different ways in which SMIs are given in practice (clinical heterogeneity). This suggests that one should explore the context under which SMIs perform better. In part, we tried to do this using CNMA and estimating component effects. Still, from an initial number of dozens of components, we had to lump them in groups so that we would have power to estimate all these effects. The problem of confounding is mitigated with CNMA because component effects are informed by the entire network as components are included in many trials. As a result, we have more precise results and a clear ordering of SMIs. On the other hand, the additivity assumption is a strong one, and we expect that components interact with each other (i.e., the effect of an SMI characteristic may differ depending on who delivers the SMI, how it is delivered and where). As a compromise between the NMA and CNMA results, we developed a series of plots that try to estimate which components work (or do not work) based on the NMA results. Pillay et al. employed a similar approach, but they made their inferences by observing which components were included in the most and least effective interventions [33]. This approach can be challenging in large networks; hence, it is recommended to use visualization tools that explore the components’ behavior and associate the presence of components with effectiveness [21]. In our research, our focus was on individual studies rather than systematic reviews. Nevertheless, there is quite an overlap between the studies included in the Pillay et al. publication and those incorporated into COMPAR-EU. For HbA1c that includes many trials, we see that the CNMA results and inferences from the graphs lead to the same conclusions (SMIs that include monitoring techniques and education, given face-to-face by professionals, produce the largest effects). Given the vast heterogeneity, more research is needed into the contextual factors that are important for an SMI’s effectiveness.

While our study strived to provide valuable insights into self-management interventions (SMIs) for Type 2 diabetes mellitus (T2DM), it is crucial to recognize the limitations of our research. Such a limitation is the timeframe of our data collection, which was restricted to studies available up to 2018. The current review was conducted as part of the COMPAR-EU study that started at that time. The field of healthcare and chronic disease management is dynamic and continuously evolving. However, based on our descriptive papers and a search of the literature, we do not expect many differences in the design and content of SMIs for diabetes in the past five years.

## 5. Conclusions

Overall, our study provides a valuable foundation for understanding the landscape of SMIs for T2DM up to 2018 and offers insights that can inform decision making and practice in the management of this chronic condition. We encourage future research to build upon our findings and consider more recent evidence to ensure the ongoing relevance and applicability of our results.

## Figures and Tables

**Table 1:** Outcomes analyzed.

Subcategory	Main Category	#Studies	#Interventions
**Basic empowerment measures**			
***Self-management competences***	Knowledge	50	35
	Self-efficacy	57	38
**Adherence to Self-management behaviors**			
	Self-management behaviors	41	30
	Adherence	55	32
	Foot care	26	28
	Glucose self-monitoring	29	26
	Dietary habits	30	29
	Consumption of fat	14	12
	Physical activity	65	47
**Clinical outcomes (and markers)**			
***Glucose management***	Glycated hemoglobin (HbA1c)	461	97
***Weight management***	Body mass index (BMI)	230	76
	Waist size	80	44
	Weight	143	60
***Blood Pressure***	Systolic blood pressure	233	71
	Diastolic blood pressure	211	68
***Lipid profile***	Low-density lipoprotein (LDL) cholesterol	171	58
	High-density lipoprotein (HDL) cholesterol	165	55
	Triglycerides	169	61
	Total cholesterol	176	68
**Quality of life**			
	Quality of life	85	42
	Psychological distress	46	39

**Table 2:** Summary of findings for all SMIs vs. usual care/usual care plus (UC/UCP).

Outcome (N of Participants; N of Studies)	Anticipated Absolute Effect (95% CI) Difference	τ^2^ (Ι^2^)	Egger’s Test	Certainty *
**HbA1c** (N = 66,280; 386 RCTs)	MD 0.39% lower (0.45 lower to 0.34 lower)	0.17 (99%)	<0.001	⨁◯◯◯ Very low
**BMI** (N = 33,574; 204 RCTs)	MD 0.28 kg/m^2^ lower (0.42 lower to 0.15 lower)	0.51 (91%)	0.03	⨁◯◯◯ Very low
**LDL cholesterol** (N = 25,580; 146 RCTs)	MD 1.78 mg/dL lower (3.02 lower to 0.53 lower)	32.94 (90%)	0.03	⨁◯◯◯ Very low

* GRADE Assessment.

**Table 3:** Subgroup analysis results for the interventions’ intensity.

Outcome	Intensity	Number of Studies	MD [95% CI]	τ^2^ (Ι^2^)
**HbA1c** (%)	High	134	−0.40 [−0.47, −0.34]	0.30 (89%)
	Low	246	−0.36 [−0.43, −0.28]	0.59 (99%)
**BMI** (kg/m^2^)	High	77	−0.39 [−0.55, −0.23]	0.50 (84%)
	Low	122	−0.14 [−0.30, −0.02]	0.67 (91%)
**LDL cholesterol** (mg/dL)	High	55	−1.71 [−2.49, −0.92]	1.08 (77%)
	Low	85	−1.23 [−1.84, −0.62]	1.17 (93%)

**Table 4:** NMA treatment effect estimates for interventions with P-score > 0.80. Statistically significant results for both 95% CI and 95% PI are in bold.

Treatment Comparison (Intervention vs. UC)	NMA Estimate MD [95% CI] (95% PI)	P-Score	% Direct Evidence
**HbA1c (%) (461 studies, 97 interventions)**			
**E + EB + SS + G**	**−1.42 [−2.02 −0.82] (−2.28, −0.57)**	**0.98**	**0%**
**E + MT + EB**	**−0.78 [−1.00, −0.57] (−1.43, −0.14)**	**0.87**	**71%**
E + G + R	−0.91 [−1.80, −0.01] (−1.99, 0.18)	0.83	0%
E + MT + P + G	−0.82 [−1.46, −0.18] (−1.71, 0.06)	0.83	54%
MT + AB + R	−0.89 [−1.73, −0.04] (−1.93, 0.16)	0.83	0%
E + AB + SS + P	−1.02 [−2.29, 0.25] (−2.43, 0.39)	0.81	0%
**E + SS + G**	**−0.69 [−1.04, −0.35] (−1.39, 0.00)**	**0.81**	**96%**
MT + R	−0.84 [−1.66, −0.02] (−1.86, 0.18)	0.81	0%
Common within-network between-study variance τ^2^ = 0.09, Ι^2^ = 86.5%			
Design-by-treatment interaction model for inconsistency X^2^ (d.f., *p*-value, τ^2^): 154.58 (128, 0.05, 0.32)			
**BMI (kg/m^2^) (230 studies, 76 interventions)**			
**E + AB + EB + SS**	**−1.88 [−2.89, −0.88] (−3.26, −0.51)**	**0.93**	**100%**
**E + MT + P + G**	**−1.70 [−3.03, −0.37] (−3.33, −0.07)**	**0.90**	**100%**
E + MT + EB + SS + G	−2.40 [−5.34, 0.54] (−5.50, 0.70)	0.89	100%
**E + MT + AB + EB + R**	**−1.28 [−1.88, −0.68] (−2.39, −0.16)**	**0.87**	**88%**
E + SD	−2.10 [−4.90, 0.70] (−5.07, 0.87)	0.86	100%
E + MT + AB + SD + P	−1.83 [−4.40, 0.74] (−4.58, 0.92)	0.84	76%
E + SS + G	−1.08 [−1.94, −0.22] (−2.35, 0.19)	0.82	100%
AB + EB	−2.80 [−8.43, 2.83] (−8.54, 2.94)	0.80	100%
Common within-network between-study variance τ^2^ = 0.22, Ι^2^ = 61.1%			
Design-by-treatment interaction model for inconsistency X^2^ (d.f., *p*-value, τ^2^): 86.19 (64, 0.03, 0.44)			
**LDL cholesterol (mg/dL) (171 studies, 58 interventions)**			
**E + MT + SS + P**	**−35.10 [−42.35, −27.84] (−42.75, −27.44)**	**1**	**0%**
**E + MT + SS + R**	**−16.63 [−21.72, −11.54] (−22.24, −11.03)**	**0.95**	**0%**
**E + AB + SD**	**−15.47 [−25.71, −5.23] (−26.05, −4.89)**	**0.92**	**100%**
**E + MT + AB + SS + R**	**−14.99 [−27.66, −2.32] (−27.98, −2.01)**	**0.89**	**0%**
**E + AB + EB + P**	**−11.44 [−14.58, −8.30] (−15.32, −7.57)**	**0.88**	**88%**
E + MT + G	−16.70 [−34.60, 1.20] (−34.91, 1.51)	0.88	100%
**E + EB + SS + G**	**−11.93 [−19.74, −4.12] (−20.13, −3.73)**	**0.87**	**0%**
**E + R**	**−10.50 [−17.66, −3.33] (−18.07, −2.92)**	**0.84**	**0%**
**E + MT + AB + SD**	**−8.77 [−11.05, −6.49] (−11.98, −5.56)**	**0.82**	**100%**
Common within-network between-study variance τ^2^ = 1.27, Ι^2^ = 70.8%			
Design-by-treatment interaction model for inconsistency X^2^ (d.f., *p*-value, τ^2^): 104.97 (46, <0.001, 2.07)			

**Table 5:** Relative effects of each component vs. UC from CNMA. Statistically significant results are denoted in bold.

Outcome	HbA1c (%)	BMI (kg/m^2^)	LDL Cholesterol (mg/dL)
**Component**	**MD [95% CI]**		
AB	0.00 [−0.08, 0.08]	−0.21 [−0.47, 0.05]	−0.47 [−1.74, 0.80]
E	**−0.25 [−0.34, −0.16]**	−0.13 [−0.44, 0.18]	**−1.84 [−3.46, −0.23]**
EB	−0.02 [−0.10, 0.05]	−0.07 [−0.31, 0.18]	**2.19 [1.06, 3.32]**
G	−0.06 [−0.17, 0.04]	0.01 [−0.29, 0.30]	−0.05 [−1.54, 1.44]
MT	**−0.14 [−0.22, −0.06]**	−0.03 [−0.29, 0.24]	0.63 [−0.82, 2.08]
P	**0.12 [0.00, 0.24]**	0.11 [−0.22, 0.44]	**−4.09 [−6.19, −1.98]**
R	−0.02 [−0.11, 0.08]	0.01 [−0.34, 0.36]	**−2.76 [−4.17, −1.36]**
SD	0.03 [−0.13, 0.19]	0.41 [−0.15, 0.98]	**−4.06 [−6.30, −1.81]**
SS	−0.06 [−0.16, 0.03]	0.05 [−0.24, 0.35]	1.19 [−0.44, 2.82]
UCP	0.00 [−0.12, 0.13]	0.12 [−0.28, 0.52]	−1.16 [−3.06, 0.75]
Common within-network between-study variance	τ^2^ = 0.13; Ι^2^ = 94.4%	τ^2^ = 0.48; Ι^2^ = 87.1%	τ^2^ = 2.61; Ι^2^ = 81.3%

## Data Availability

All results from analyses for all the outcomes are available in the manuscript and the COMPAR-EU platform (https://platform.self-management.eu).

## Supplementary Materials

The following supporting information can be downloaded at: https://www.mdpi.com/article/10.3390/healthcare12010027/s1, Supplementary Material S1: PRISMA-NMA checklist; Supplementary Material S2: Search strategy, inclusion criteria and flow chart; Supplementary Material S3: MA results; Supplementary Material S4: Funnel plots; Supplementary Material S5: NMA results; Supplementary Material S6: Meta-regression results; Supplementary Material S7: Subgroup analysis results; Supplementary Material S8: CNMA results; Supplementary Material S9: RoB assessment; Supplementary Material S10: CINeMA results.
