# Supplementary material for: Exploring the Effectiveness of Self-Management Interventions in Type 2 Diabetes: A Systematic Review and Network Meta-Analysis
Source: Healthcare (Basel). 2023 Dec 22;12(1):27. doi: 10.3390/healthcare12010027 (PMC10779199; doi:10.3390/healthcare12010027)
Supplement: Supplementary file 1 [file healthcare-12-00027-s001.zip › Supplementary Materials 18DEC2023.zip!/Supplementary Material S2-Search Strategy, 坣clusion Criteria and Flow chart.pdf]

Appendix A. Search Strategy, Inclusion Criteria & Flow chart

Search Strategy

PubMed (Search Date December 5th, 2018)

Search	*Query*	*Items found*
#3	#1 AND #2 AND #3 Filters: Publication date from 2010/01/01	6988
#3	("Clinical Trials as Topic"[Mesh] OR "Randomized Controlled Trial" [pt] OR "Controlled Clinical Trial" [pt] OR randomized [tiab] OR randomly[tiab] OR placebo[tiab] OR trial[ti]) NOT (animals[mh] NOT humans[mh])	1166396
#2	"Self Care"[Mesh] OR "Self-Management"[Mesh] OR "Power (Psychology)"[Mesh] OR "Health Education"[Mesh] OR "Patient Participation"[Mesh] OR "Decision Making"[Mesh] OR "Telemedicine"[Mesh] OR self-administration*[tiab] OR self-care[tiab] OR self efficac*[tiab] OR self-manag*[tiab] OR selfmanag*[tiab] OR self-monitor*[tiab] OR selfmonitor*[tiab] OR self-diagnos*[tiab] OR selfdiagnos*[tiab] OR self-assess*[tiab] OR selfassess*[tiab] OR self-direct*[tiab] OR selfdirect*[tiab] OR self-help*[tiab] OR empower*[tiab] OR enablement[tiab] OR health education[tiab] OR patient education[tiab] OR patient participation[tiab] OR coach*[tiab] OR health promot*[tiab] OR ((community[tiab] OR peer[tiab]) AND (support*[tiab] OR advice*[tiab] OR monitor*[tiab] OR train*[tiab] OR instruction*[tiab] OR intervention*[tiab] OR consult*[tiab] OR assist*[tiab] OR educat*[tiab] OR information*[tiab] OR skill*[tiab])) OR group support*[tiab] OR group intervention*[tiab] OR group advice*[tiab] OR group monitor*[tiab] OR group train*[tiab] OR training group*[tiab] OR group instruct*[tiab] OR group assist*[tiab] OR group educat*[tiab] OR group inform*[tiab] OR group skill*[tiab] OR ((patient[tiab] OR patients[tiab]) AND (centered[tiab] OR centred[tiab] OR focus*[tiab] of directed[tiab] OR coach*[tiab] OR engage*[tiab] OR involve*[tiab] OR orient*[tiab] OR participat*[tiab])) OR ((patient[tiab] OR patients[tiab]) AND (educat*[tiab] OR train*[tiab] OR instruct*[tiab] OR teach*[tiab])) OR (management*[tiab] AND (plan[tiab] OR program*[tiab] OR disease[tiab])) OR (symptom*[tiab] AND (management[tiab] OR directed[tiab] OR focus*[tiab])) OR ((personalized[tiab] OR personalized[tiab]) AND care[tiab]) OR telemedicine[tiab] OR eHealth[tiab] OR e-Health[tiab] OR mHealth[tiab] OR m-Health[tiab] OR shared decision*[tiab] OR sharing decision*[tiab] OR informed decision*[tiab] OR informed choice*[tiab] or decision aid*[tiab] OR ((share*[ti] OR sharing*[ti] OR informed*[ti]) AND (decision*[ti] OR deciding*[ti] OR choice*[ti]))	1902834
#1	"Diabetes Mellitus"[Mesh] OR diabet*[tiab]	626033

Embase (Search Date December 5th, 2018)

Search	*Query*	*Items found*
	Voor export #6 not pmids	2602
#6	#5 NOT ('chapter'/it OR 'conference abstract'/it OR 'conference review'/it OR 'editorial'/it OR 'letter'/it OR 'note'/it OR 'short survey'/it)	5322

Search	*Query*	*Items found*
#5	#1 AND #2 AND #3 AND [2010-2019]/py	8004
#4	#1 AND #2 AND #3	10233
#3	('clinical trial (topic)'/exp OR randomized:ab,ti,kw OR randomly:ab,ti,kw OR placebo:ab,ti,kw OR trial:ti) NOT ([animals]/lim NOT [humans]/lim)	1266571
#2	'self care'/exp OR 'coping behavior'/exp OR 'drug self administration'/exp OR 'self monitoring'/exp OR 'empowerment'/exp OR 'health education'/exp OR 'patient participation'/exp OR 'shared decision making'/exp OR 'telehealth'/exp OR 'self administration*':ab,ti,kw OR 'self care':ab,ti,kw OR 'self efficac*':ab,ti,kw OR 'self manag*':ab,ti,kw OR selfmanag*:ab,ti,kw OR 'self monitor*':ab,ti,kw OR selfmonitor*:ab,ti,kw OR 'self diagnos*':ab,ti,kw OR selfdiagnos*:ab,ti,kw OR 'self assess*':ab,ti,kw OR selfassess*:ab,ti,kw OR 'self direct*':ab,ti,kw OR selfdirect*:ab,ti,kw OR 'self help*':ab,ti,kw OR empower*:ab,ti,kw OR enablement:ab,ti,kw OR 'health education':ab,ti,kw OR 'patient education':ab,ti,kw OR 'patient participation':ab,ti,kw OR coach*:ab,ti,kw OR 'health promot*':ab,ti,kw OR (((group OR peer) NEAR/3 (support* OR advice* OR monitor* OR intervention* OR train* OR instruction* OR consult* OR assist* OR educat* OR information* OR skill*)):ab,ti,kw) OR (((patient OR patients) NEAR/3 (centered OR centred OR focus* OR directed OR coach* OR engage* OR involve* OR orient* OR participat*)):ab,ti,kw) OR (((patient OR patients) NEAR/3 (educat* OR train* OR instruct* OR teach*)):ab,ti,kw) OR ((management* NEAR/3 (plan OR program* OR disease)):ab,ti,kw) OR ((symptom* NEAR/3 (management OR directed OR focus*)):ab,ti,kw) OR (((personalized OR personalized) NEAR/3 care):ab,ti,kw) OR telemedicine:ab,ti,kw OR ehealth:ab,ti,kw OR 'e health':ab,ti,kw OR mhealth:ab,ti,kw OR 'm health':ab,ti,kw OR 'shared decision*':ab,ti,kw OR 'sharing decision*':ab,ti,kw OR 'informed decision*':ab,ti,kw OR 'informed choice*':ab,ti,kw OR 'decision aid*':ab,ti,kw OR (((share* OR sharing* OR informed*) NEAR/3 (decision* OR deciding* OR choice*)):ab,ti,kw)	971257
#1	'diabetes mellitus'/exp OR diabet*:ab,ti,kw	1041731

Embase and psycINFO (Search Date December 5th, 2018)

Search	*Query*	*Items found*
S5	S1 AND S2 AND S3<br>**Limiters** - Publication Year: 2010-2018	826
S4	S1 AND S2 AND S3	1186
S3	DE "Clinical Trials" OR TI (randomized OR randomly OR placebo OR trial) OR AB (randomized OR randomly OR placebo) OR KW (randomized OR randomly OR placebo OR trial) NOT (PO Animal NOT PO Human)	165196

Search	*Query*	*Items found*
S2	DE ("Self-Care Skills" OR "Self-Efficacy" OR "Drug Self Administration" OR "Coping Behavior" OR "Empowerment" OR "Telemedicine" OR "Health Education" OR "Health Promotion" OR "Client Participation") OR TI (“self administration*” OR “self care” OR “self efficac*” OR “self manag*” OR selfmanag* OR “self monitor*” OR selfmonitor* OR “self diagnos*” OR selfdiagnos* OR “self assess*” OR selfassess* OR “self direct*” OR selfdirect* OR “self help*” OR empower* OR enablement OR “health education” OR “patient education” OR “patient participation” OR coach* OR “health promot*” OR (((group OR peer) N3 (support* OR advice* OR monitor* OR intervention* OR train* OR instruction* OR consult* OR assist* OR educat* OR information* OR skill*))) OR (((patient OR patients) N3 (centered OR centred OR focus* OR directed OR coach* OR engage* OR involve* OR orient* OR participat*))) OR (((patient OR patients) N3 (educat* OR train* OR instruct* OR teach*))) OR ((management* N3 (plan OR program* OR disease))) OR ((symptom* N3 (management OR directed OR focus*))) OR (((personalized OR personalized) N3 care)) OR telemedicine OR ehealth OR “e health” OR mhealth OR “m health” OR “shared decision*” OR “sharing decision*” OR “informed decision*” OR “informed choice*” OR “decision aid*” OR (((share* OR sharing* OR informed*) N3 (decision* OR deciding* OR choice*))) OR AB (“self administration*” OR “self care” OR “self efficac*” OR “self manag*” OR selfmanag* OR “self monitor*” OR selfmonitor* OR “self diagnos*” OR selfdiagnos* OR “self assess*” OR selfassess* OR “self direct*” OR selfdirect* OR “self help*” OR empower* OR enablement OR “health education” OR “patient education” OR “patient participation” OR coach* OR “health promot*” OR (((group OR peer) N3 (support* OR advice* OR monitor* OR intervention* OR train* OR instruction* OR consult* OR assist* OR educat* OR information* OR skill*))) OR (((patient OR patients) N3 (centered OR centred OR focus* OR directed OR coach* OR engage* OR involve* OR orient* OR participat*))) OR (((patient OR patients) N3 (educat* OR train* OR instruct* OR teach*))) OR ((management* N3 (plan OR program* OR disease))) OR ((symptom* N3 (management OR directed OR focus*))) OR (((personalized OR personalized) N3 care)) OR telemedicine OR ehealth OR “e health” OR mhealth OR “m health” OR “shared decision*” OR “sharing decision*” OR “informed decision*” OR “informed choice*” OR “decision aid*” OR (((share* OR sharing* OR informed*) N3 (decision* OR deciding* OR choice*))) OR KW (“self administration*” OR “self care” OR “self efficac*” OR “self manag*” OR selfmanag* OR “self monitor*” OR selfmonitor* OR “self diagnos*” OR selfdiagnos* OR “self assess*” OR selfassess* OR “self direct*” OR selfdirect* OR “self help*” OR empower* OR enablement OR “health education” OR “patient education” OR “patient participation” OR coach* OR “health promot*” OR (((group OR peer) N3 (support* OR advice* OR monitor* OR intervention* OR train* OR instruction* OR consult* OR assist* OR educat* OR information* OR skill*))) OR (((patient OR patients) N3 (centered OR centred OR focus* OR directed OR coach* OR engage* OR involve* OR orient* OR participat*))) OR (((patient OR patients) N3 (educat* OR train* OR instruct* OR teach*))) OR ((management* N3 (plan OR program* OR disease))) OR ((symptom* N3 (management OR directed OR focus*))) OR (((personalized OR personalized) N3 care)) OR telemedicine OR ehealth OR “e health” OR mhealth OR “m health” OR “shared decision*” OR “sharing decision*” OR “informed decision*” OR “informed choice*” OR “decision aid*” OR (((share* OR sharing* OR informed*) N3 (decision* OR deciding* OR choice*)))	410390
S1	DE ("Diabetes Mellitus" OR "Type 2 Diabetes") OR TI diabet* OR AB diabet* OR KW diabet*	29185

Cinahl (Search Date December 5th, 2018)

Search	*Query*	*Items found*
S5	S1 AND S2 AND S3<br>**Limiters** - Publication Year: 2010-2018, academic journals	2229
S4	S1 AND S2 AND S3	4310
S3	MH "Clinical Trials+" OR TI (randomized OR randomly OR placebo OR trial) OR AB (randomized OR randomly OR placebo) NOT (MH "Animals" NOT MH "Human)	377603
S2	MH ("Self Care+" OR "Self-Efficacy" OR "Coping" OR"Empowerment" OR "Telehealth+") OR "Health Education" OR "Consumer Participation") OR TI (“self administration*” OR “self care” OR “self efficac*” OR “self manag*” OR selfmanag* OR “self monitor*” OR selfmonitor* OR “self diagnos*” OR selfdiagnos* OR “self assess*” OR selfassess* OR “self direct*” OR selfdirect* OR “self help*” OR empower* OR enablement OR “health education” OR “patient education” OR “patient participation” OR coach* OR “health promot*” OR (((group OR peer) N3 (support* OR advice* OR monitor* OR intervention* OR train* OR instruction* OR consult* OR assist* OR educat* OR information* OR skill*))) OR (((patient OR patients) N3 (centered OR centred OR focus* OR directed OR coach* OR engage* OR involve* OR orient* OR participat*))) OR (((patient OR patients) N3 (educat* OR train* OR instruct* OR teach*))) OR ((management* N3 (plan OR program* OR disease))) OR ((symptom* N3 (management OR directed OR focus*))) OR (((personalized OR personalized) N3 care)) OR telemedicine OR ehealth OR “e health” OR mhealth OR “m health” OR “shared decision*” OR “sharing decision*” OR “informed decision*” OR “informed choice*” OR “decision aid*” OR (((share* OR sharing* OR informed*) N3 (decision* OR deciding* OR choice*))) OR AB (“self administration*” OR “self care” OR “self efficac*” OR “self manag*” OR selfmanag* OR “self monitor*” OR selfmonitor* OR “self diagnos*” OR selfdiagnos* OR “self assess*” OR selfassess* OR “self direct*” OR selfdirect* OR “self help*” OR empower* OR enablement OR “health education” OR “patient education” OR “patient participation” OR coach* OR “health promot*” OR (((group OR peer) N3 (support* OR advice* OR monitor* OR intervention* OR train* OR instruction* OR consult* OR assist* OR educat* OR information* OR skill*))) OR (((patient OR patients) N3 (centered OR centred OR focus* OR directed OR coach* OR engage* OR involve* OR orient* OR participat*))) OR (((patient OR patients) N3 (educat* OR train* OR instruct* OR teach*))) OR ((management* N3 (plan OR program* OR disease))) OR ((symptom* N3 (management OR directed OR focus*))) OR (((personalized OR personalized) N3 care)) OR telemedicine OR ehealth OR “e health” OR mhealth OR “m health” OR “shared decision*” OR “sharing decision*” OR “informed decision*” OR “informed choice*” OR “decision aid*” OR (((share* OR sharing* OR informed*) N3 (decision* OR deciding* OR choice*)))	472850
S1	MH "Diabetes Mellitus+" OR TI diabet* OR AB diabet*	186369

Search History Cochrane Central Trial Register (Search Date December 5th, 2018)

Search	*Query*	*Items found*
#4	#3 Limits Publication Year from 2010 to 2018, with Cochrane Library publication date from Jan 2010 to Dec 2018,	7386
#3	#1 AND #2	
#2	(self NEXT administration* OR self NEXT care OR self NEXT efficac* OR self NEXT manag* OR selfmanag* OR self NEXT monitor* OR selfmonitor* OR self NEXT diagnos* OR selfdiagnos* OR self NEXT assess* OR selfassess* OR self NEXT direct* OR selfdirect* OR self NEXT help* OR empower* OR enablement OR health NEXT education OR patient NEXT education OR patient NEXT participation OR coach* OR health NEXT promot* OR (((group OR peer) NEAR/3 (support* OR advice* OR monitor* OR intervention* OR train* OR instruction* OR consult* OR assist* OR educat* OR information* OR skill*))) OR (((patient OR patients) NEAR/3 (centered OR centred OR focus* OR directed OR coach* OR engage* OR involve* OR orient* OR participat*)) OR (((patient OR patients) NEAR/3 (educat* OR train* OR instruct* OR teach*)) OR ((management* NEAR/3 (plan OR program* OR disease))) OR ((symptom* NEAR/3 (management OR directed OR focus*))) OR (((personalized OR personalized) NEAR/3 care)) OR telemedicine OR ehealth OR "e-health" OR mhealth OR "m-health" OR shared NEXT decision* OR sharing NEXT decision* OR informed NEXT decision* OR informed NEXT choice* OR decision NEXT aid* OR (((share* OR sharing* OR informed*) NEAR/3 (decision* OR deciding* OR choice*)))):ab,ti,kw	120971
#1	diabet*:ab,ti,kw	64482

<u>Inclusion Criteria</u>

Population	Adults (>18 years) with Diabetes Type-II (T2DM)
Intervention	Self-management support techniques, types of support, mode of delivery, type of provider, intensity
Comparison	Usual care or any self-management interventions
Outcomes	Not to be specified at the level of literature search
Study design	RCTs (quasi-randomized studies were excluded)

Identification of studies via databases and registers

Identification

11780 records identified.

10125 records were excluded.

Screening

1657 records screened.

992 records excluded:

- Invalid article type = 137
- No RCTs = 139
- No DMT2 = 45
- Mixed population = 79
- Wrong publication = 14
- No SMI = 259
- SMI not main focus = 43
- No outcome of interest = 135
- Duplicates = 16
- Multiple publication = 125

665 studies obtained.

#Studies obtained per outcome:

Knowledge = 114, Self-efficacy = 160,
Self-management behaviours = 72,
Adherence = 97, Foot care = 49,
Glucose Self-monitoring = 59,
Dietary habits = 67, Consumption of fat = 27,
Physical activity = 135, Glycated haemoglobin = 585,
Body Mass Index = 286, Waist size = 112,
Weight = 192, Systolic blood pressure = 296,
Diastolic blood pressure = 271,
Low-density lipoprotein cholesterol = 238,
High-density lipoprotein cholesterol = 225,
Triglycerides = 223, Total cholesterol = 239,
Quality of life = 182, Psychological distress = 89

#Studies excluded due to missing values:

Knowledge = 64, Self-efficacy =103,
Self-management behaviours = 31,
Adherence = 42, Foot care = 23,
Glucose Self-monitoring = 30,
Dietary habits = 37, Consumption of fat = 13,
Physical activity = 70, Glycated haemoglobin = 124,
Body Mass Index = 56, Waist size = 32,
Weight = 49, Systolic blood pressure = 63,
Diastolic blood pressure = 60,
Low-density lipoprotein cholesterol = 67,
High-density lipoprotein cholesterol = 60,
Triglycerides = 54, Total cholesterol = 63,
Quality of life = 97, Psychological distress = 43

Included

#Studies included in the analysis:

Knowledge = 50, Self-efficacy = 57,
Self-management behaviours = 41,
Adherence = 55, Foot care = 26,
Glucose Self-monitoring = 29,
Dietary habits = 30, Consumption of fat = 14,
Physical activity = 65, Glycated haemoglobin = 461,
Body Mass Index = 230, Waist size = 80,
Weight = 143, Systolic blood pressure = 233,
Diastolic blood pressure = 211,
Low-density lipoprotein cholesterol = 171,
High-density lipoprotein cholesterol = 165,
Triglycerides = 169, Total cholesterol = 176,
Quality of life = 85, Psychological distress = 46
