# Supplementary material for: Exploring the Effectiveness of Self-Management Interventions in Type 2 Diabetes: A Systematic Review and Network Meta-Analysis
Source: Healthcare (Basel). 2023 Dec 22;12(1):27. doi: 10.3390/healthcare12010027 (PMC10779199; doi:10.3390/healthcare12010027)
Supplement: Supplementary file 1 [file healthcare-12-00027-s001.zip › Supplementary Materials 18DEC2023.zip!/Supplementary Material S3-MA Results.pdf]

Appendix B. Meta-Analysis Results (Fixed- and Random-effects models)

Outcome	# Studies	Fixed-effects TE [95% CI]	Random-effects TE [95% CI]	τ^2 (I^2)	Prediction Interval	P-value for Egger's test
Knowledge	41	SMD 0.73 [0.67, 0.79]	SMD 0.92 [0.69, 1.16]	0.49 (92%)	[-0.51, 2.35]	0.002
Adherence	49	SMD 0.14 [0.09, 0.19]	SMD 0.34 [0.10, 0.57]	0.34 (91%)	[-0.88, 1.55]	0.02
Body Mass Index (kg/m^2)	204	MD -0.01 [-0.04, 0.01]	MD -0.28 [-0.41, -0.15]	0.51 (91%)	[-1.69, 1.13]	0.03
Care Satisfaction	12	SMD 0.44 [0.35, 0.52]	SMD 0.60 [0.00, 1.20]	0.79 (95%)	[-1.46, 2.67]	0.45
Consumption of Fat	12	MD -0.41 [-0.50, -0.32]	MD -0.43 [-0.76, -0.10]	0.26 (89%)	[-1.60, 0.75]	0.76
Diastolic Blood Pressure (mmHg)	178	MD -1.33 [-1.44, -1.22]	MD -1.14 [-1.50, -0.78]	3.09 (86%)	[-4.63, 2.35]	0.33
Dietary Habits	26	SMD 0.30 [0.23, 0.37]	SMD 0.33 [0.10, 0.56]	0.25 (83%)	[-0.73, 1.39]	0.53
Footcare	22	SMD 0.68 [0.61, 0.76]	SMD 0.78 [0.38, 1.18]	0.73 (95%)	[-1.05, 2.61]	0.43
Glucose Self-monitoring	24	SMD 0.39 [0.32, 0.46]	SMD 0.57 [0.23, 0.92]	0.58 (90%)	[-1.04, 2.19]	0.04
HbA1c (%)	386	MD 0.04 [0.03, 0.04]	MD-0.39 [-0.44, -0.34]	0.17 (99%)	[-1.20, 0.42]	<0.001
HDL cholesterol (mg/dL)	136	MD 0.00 [-0.02, 0.01]	MD 0.27 [-0.16, 0.69]	3.67 (76%)	[-3.54, 4.08]	0.33
LDL cholesterol (mg/dL)	146	MD -0.07 [-0.12, -0.01]	MD -1.78 [-3.02, -0.53]	32.94 (90%)	[-13.19, 9.63]	0.03
Physical Activity	49	SMD 0.28 [0.23, 0.33]	SMD 0.40 [0.26, 0.54]	0.18 (81%)	[-0.46, 1.25]	0.002
Psychological Distress	31	SMD 0.03 [-0.02, 0.08]	SMD 0.08 [-0.12, 0.29]	0.21 (82%)	[-0.88, 1.05]	0.63
Quality of Life	73	SMD 0.15 [0.11, 0.19]	SMD 0.24 [0.07, 0.41]	0.47 (90%)	[-1.14, 1.61]	0.09
Self-efficacy	53	SMD 0.27 [0.22, 0.31]	SMD 0.37 [0.17, 0.57]	0.49 (92%)	[-1.05, 1.79]	0.11
Self-management Behaviours	36	SMD 0.50 [0.44, 0.56]	SMD 0.63 [0.44, 0.82]	0.28 (89%)	[-0.46, 1.71]	0.01
Systolic Blood Pressure (mmHg)	199	MD -2.90 [-3.10, -2.70]	MD -2.04 [-2.60, -1.48]	8.45 (80%)	[-7.80, 3.72]	0.005
Total Cholesterol (mg/dL)	149	MD 0.14 [0.08, 0.19]	MD -3.01 [-4.71, -1.31]	74.14 (96%)	[-20.10, 14.09]	0.06
Triglycerides (mg/dL)	145	MD -0.01 [-0.02, -0.00]	MD -0.12 [-0.20, -0.04]	0.13 (89%)	[-0.83, 0.59]	0.02
Waist size (cm)	71	MD -0.69 [-0.87, -0.52]	MD -1.39 [-1.97, -0.80]	2.3 (66%)	[-4.47, 1.70]	0.002
Weight (kg)	115	MD -0.59 [-0.62, -0.57]	MD -0.98 [-1.45, -0.51]	2.68 (74%)	[-4.26, 2.30]	0.16
