# Supplementary material for: Exploring the Effectiveness of Self-Management Interventions in Type 2 Diabetes: A Systematic Review and Network Meta-Analysis
Source: Healthcare (Basel). 2023 Dec 22;12(1):27. doi: 10.3390/healthcare12010027 (PMC10779199; doi:10.3390/healthcare12010027)

Appendix C. Funnel Plots

a. **Knowledge**

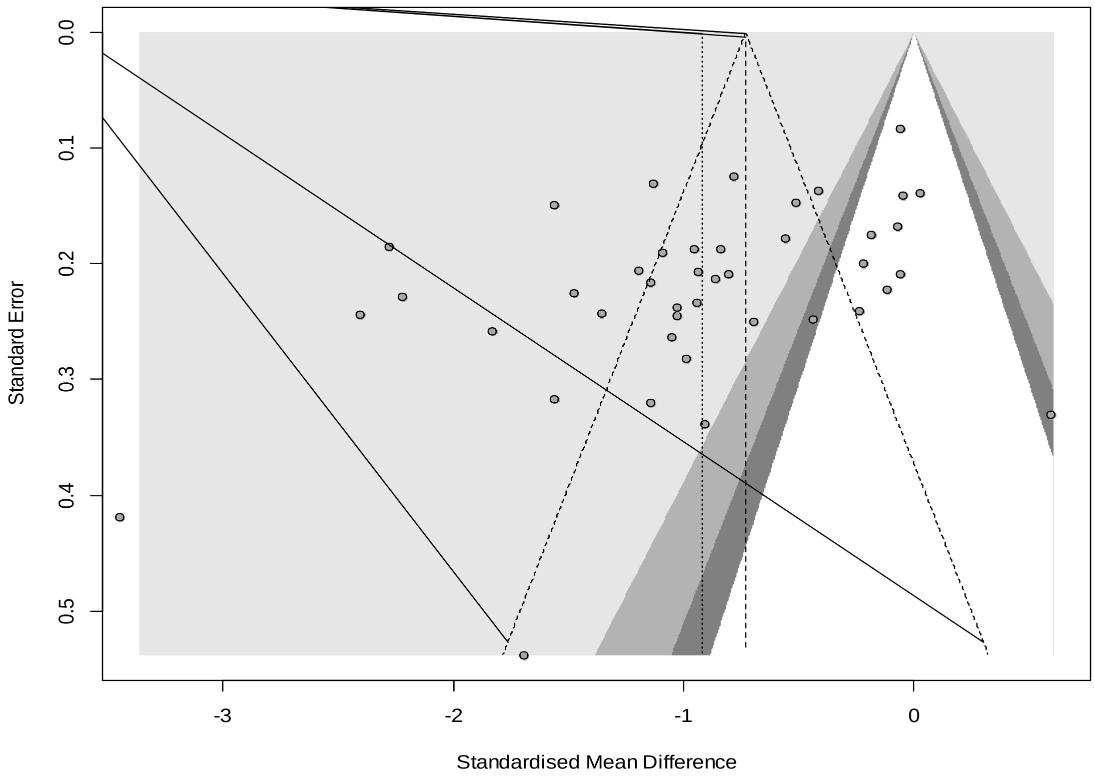

b. **Self-efficacy**

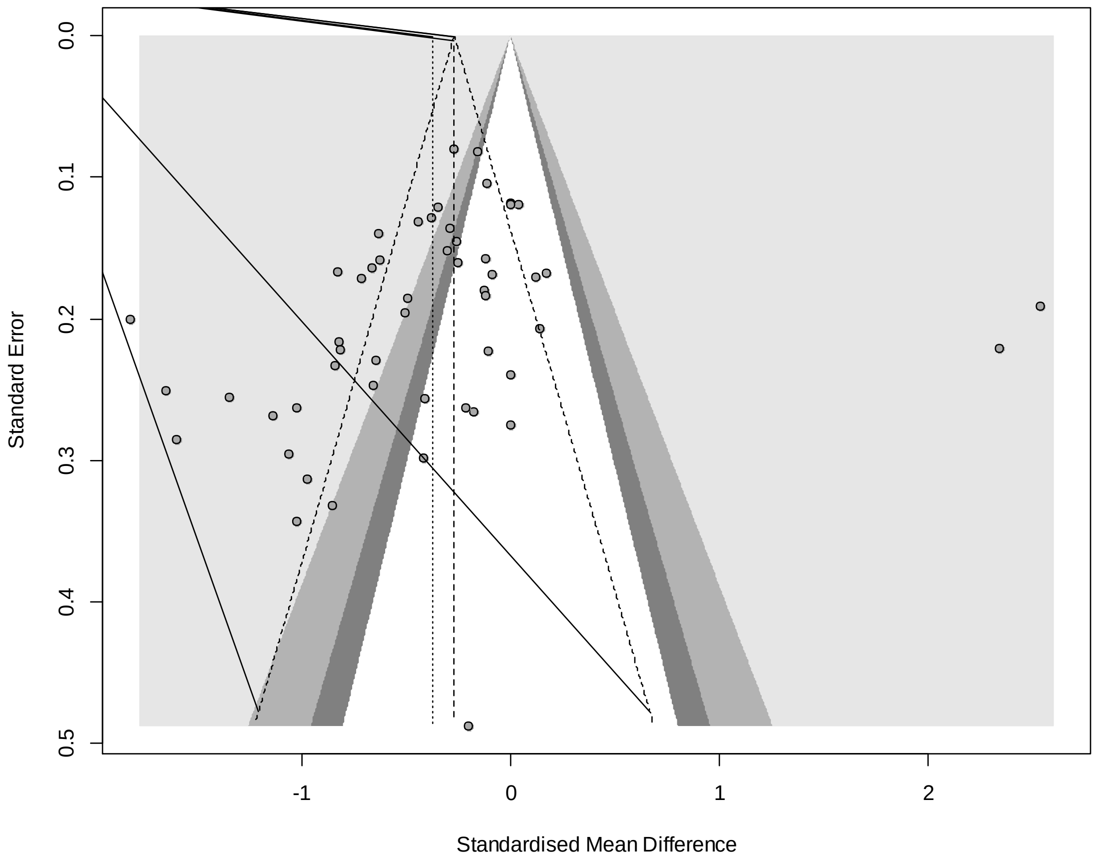

c. Self-management behaviours

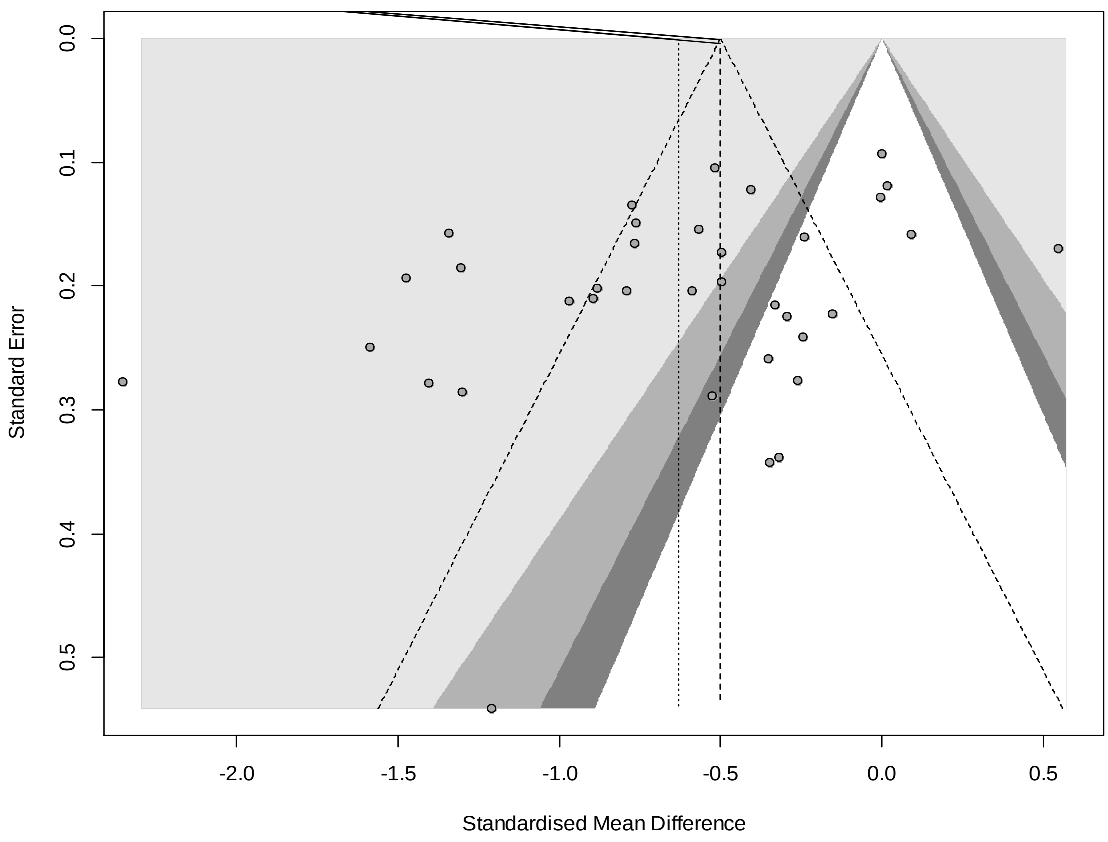

d. Adherence

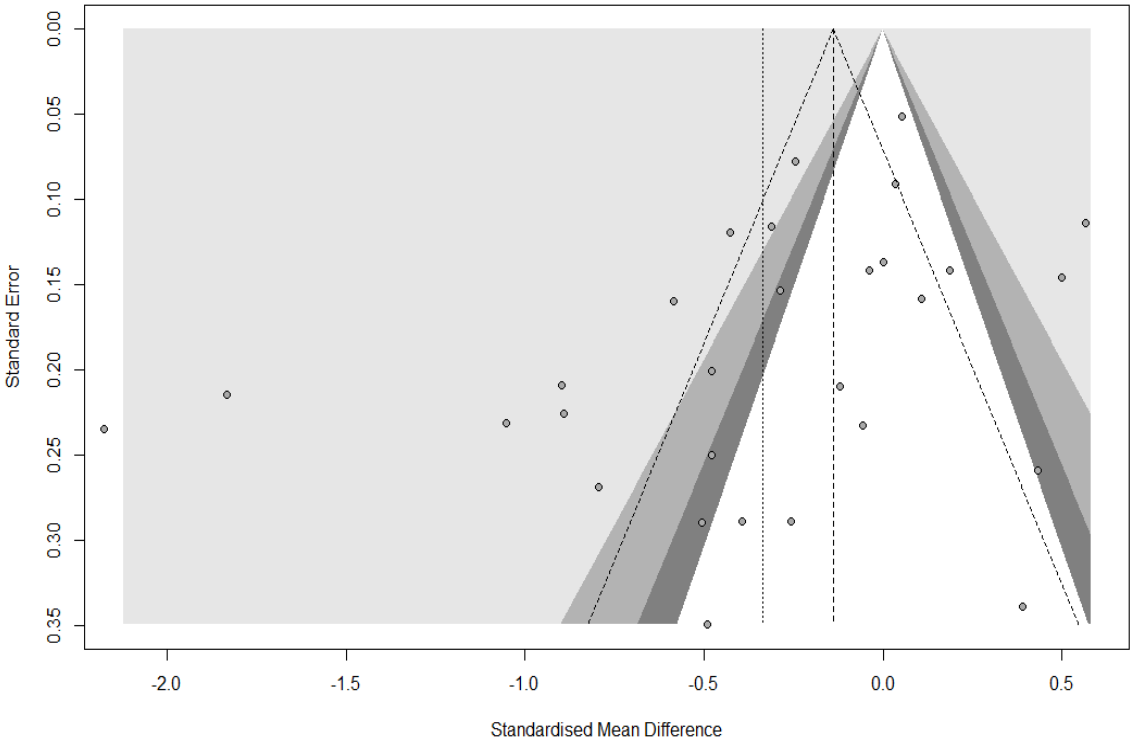

e. Foot care

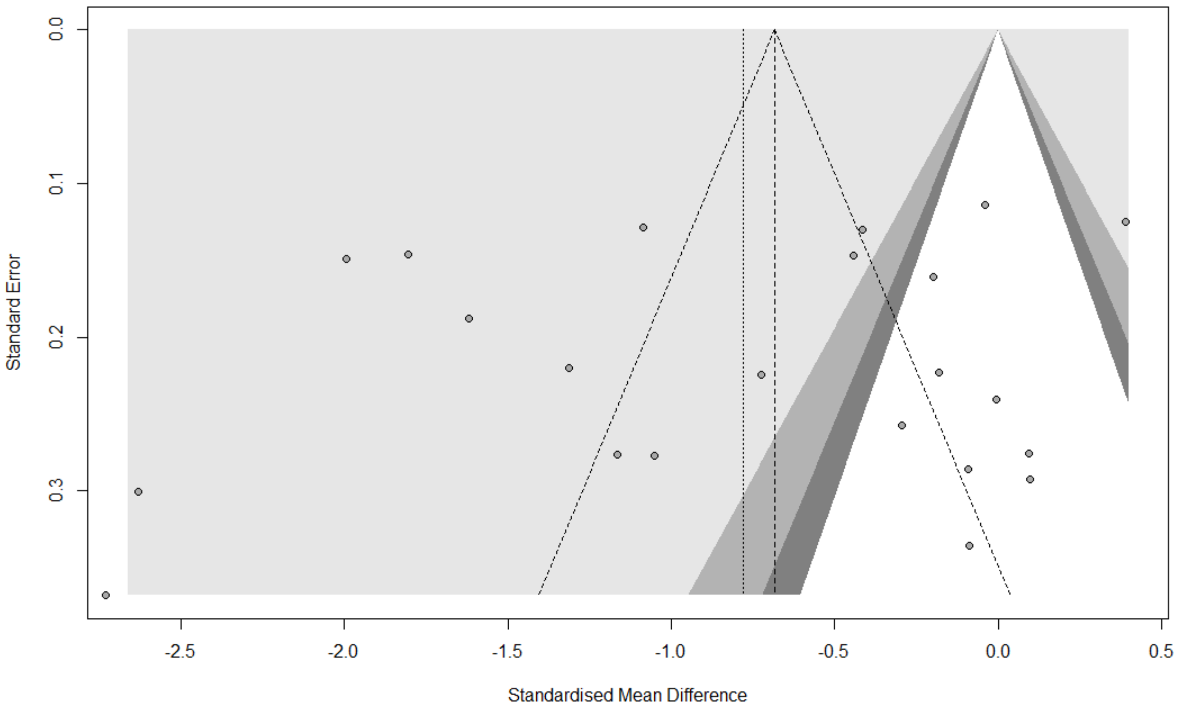

f. Glucose Self-monitoring

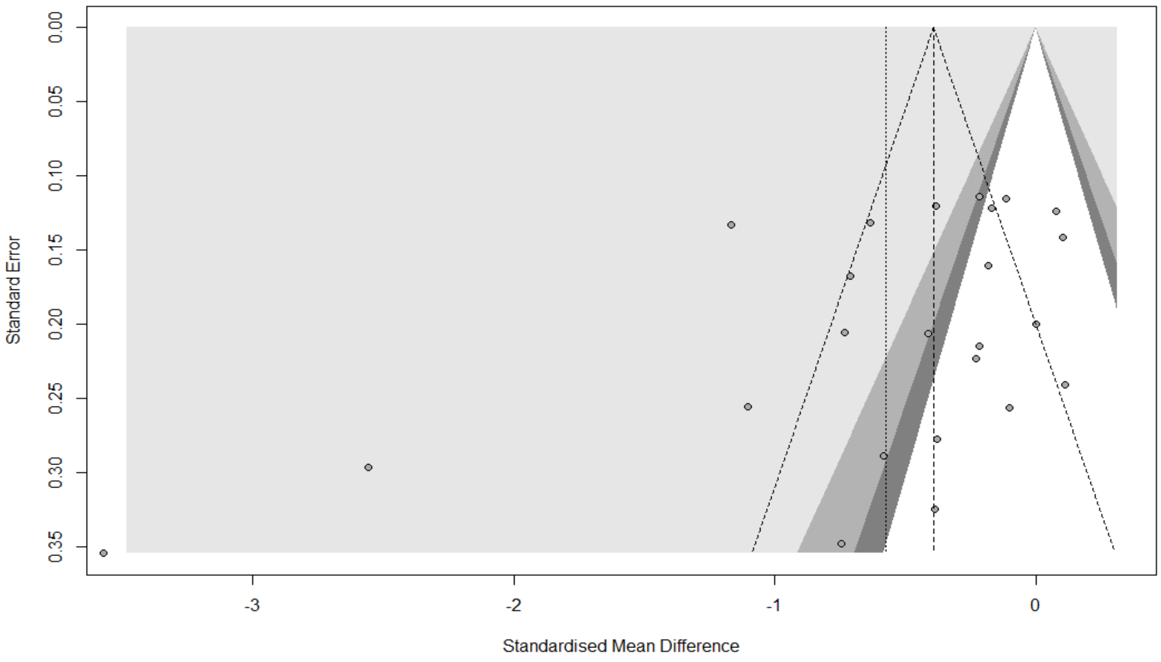

g. Dietary habits

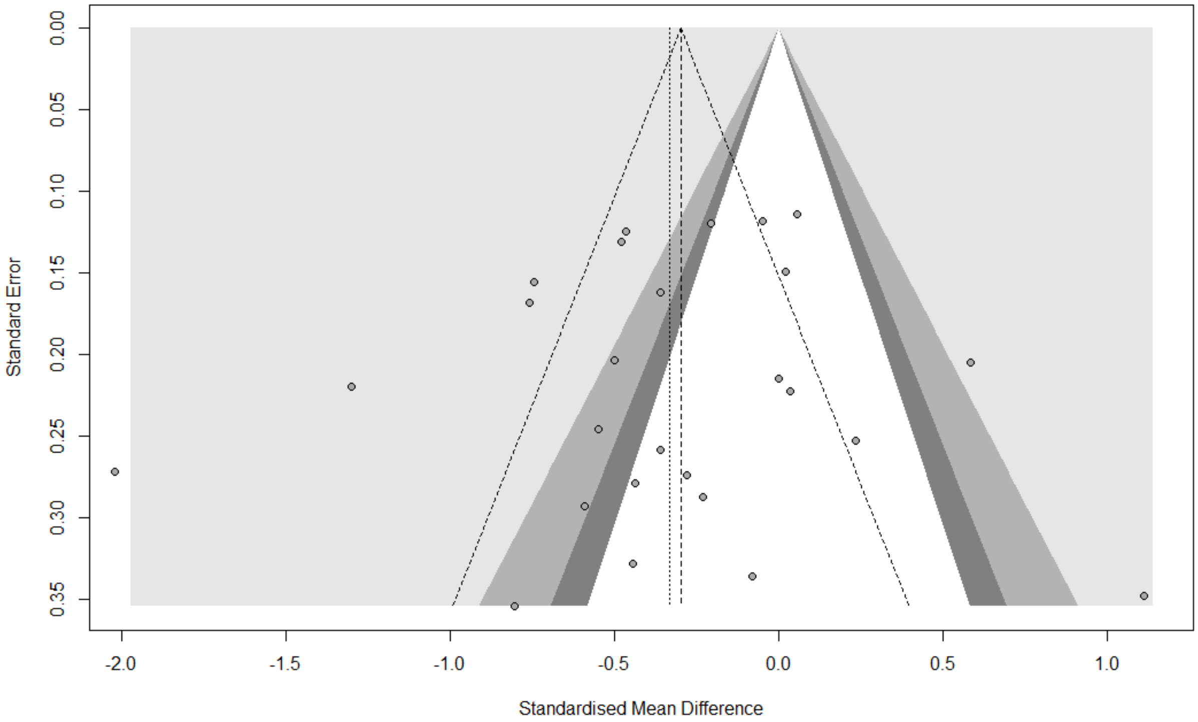

h. Consumption of fat

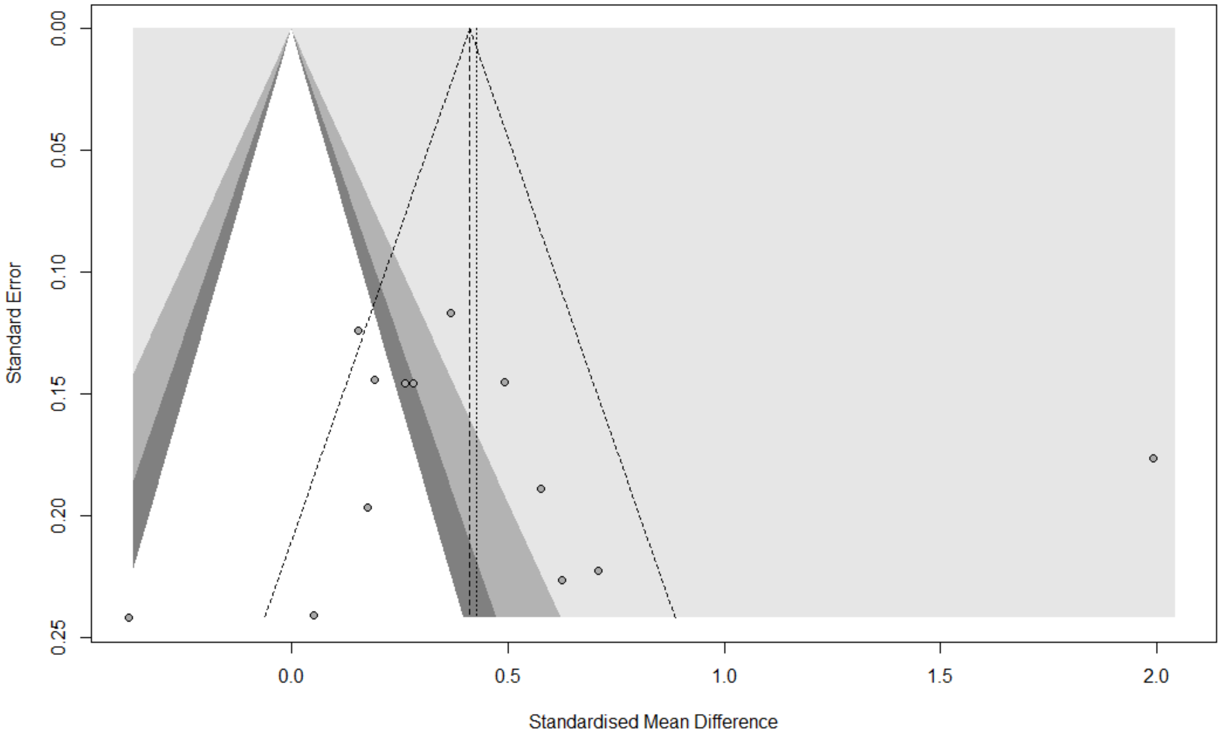

i. Physical activity

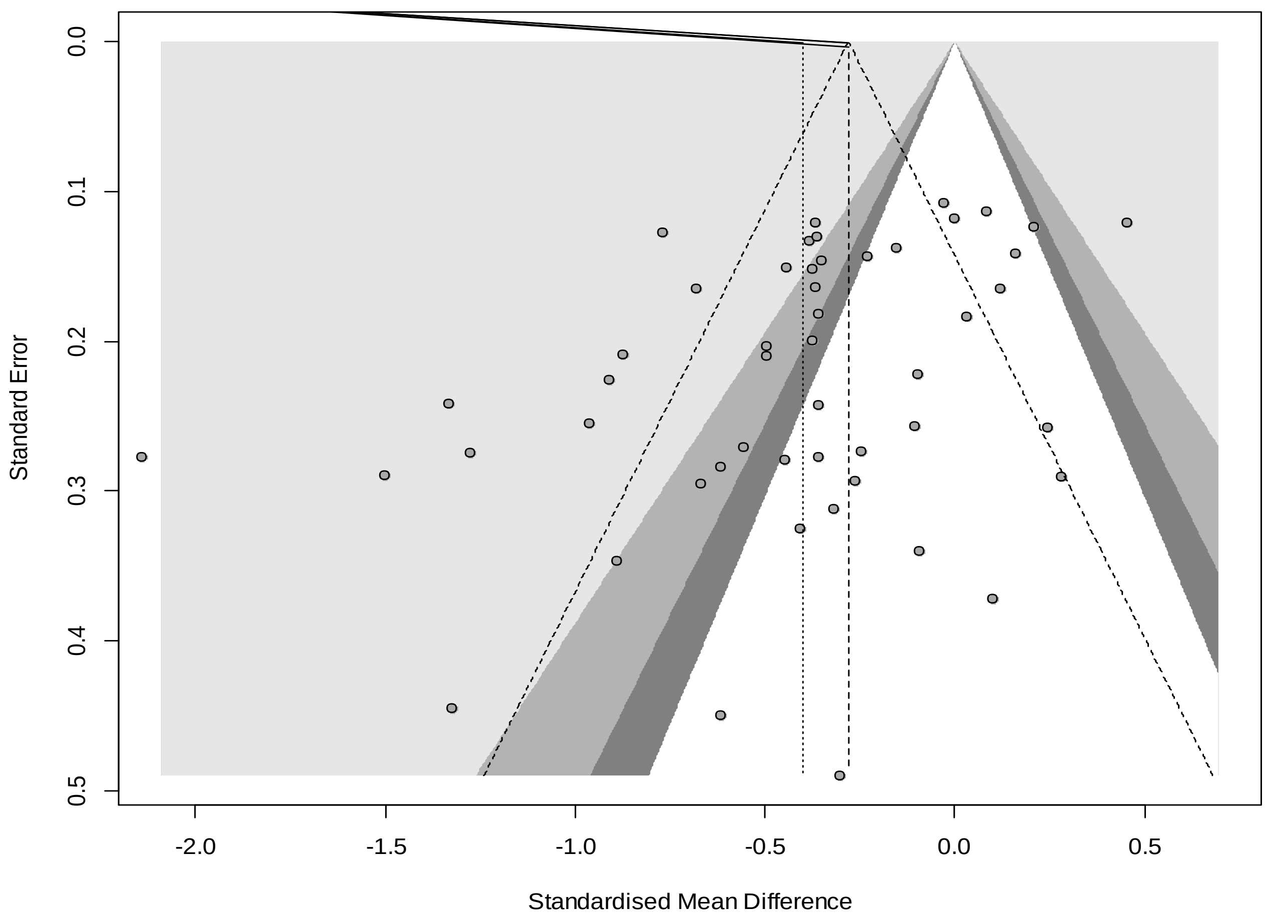

j. Glycated haemoglobin (HbA1c)

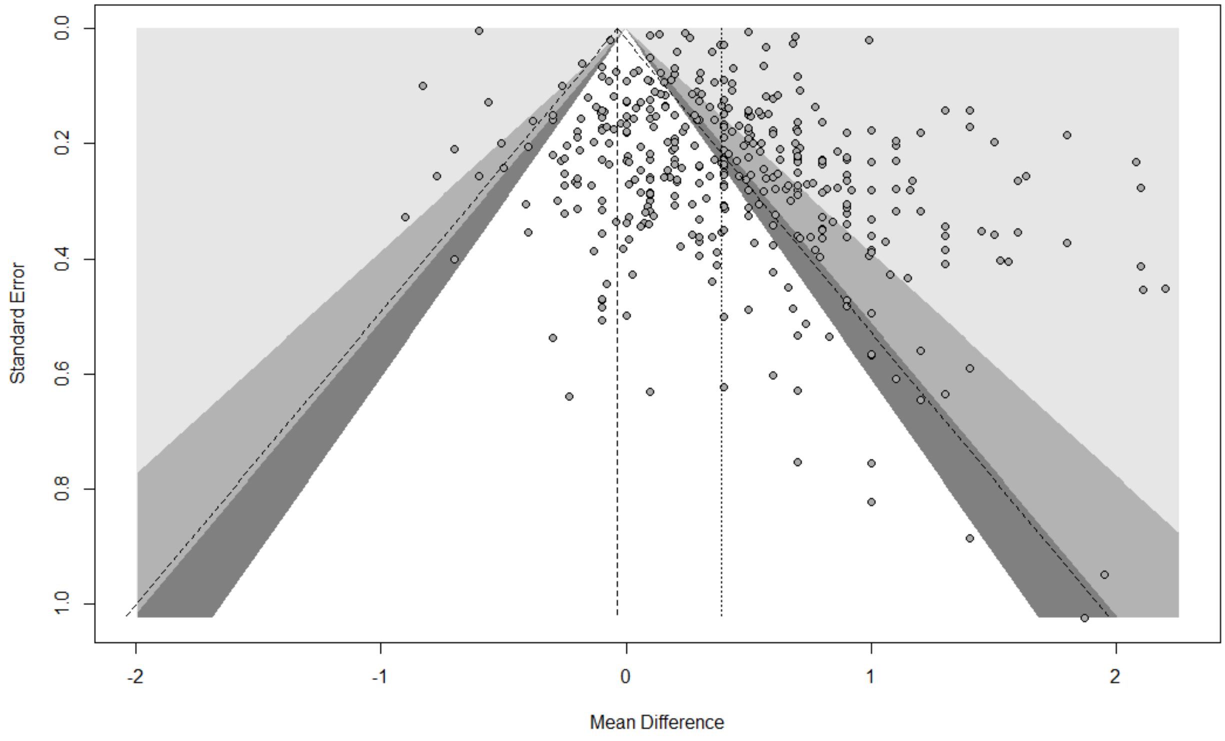

k. Body Mass Index (BMI)

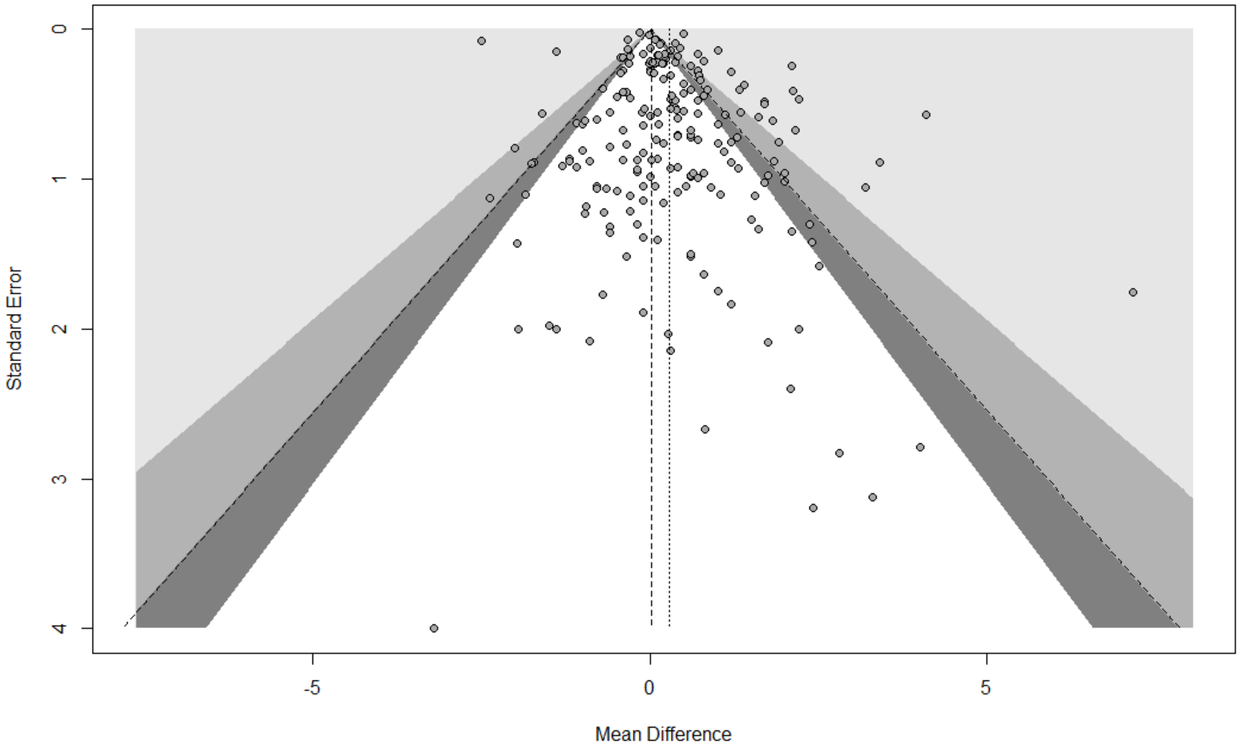

l. Waist size

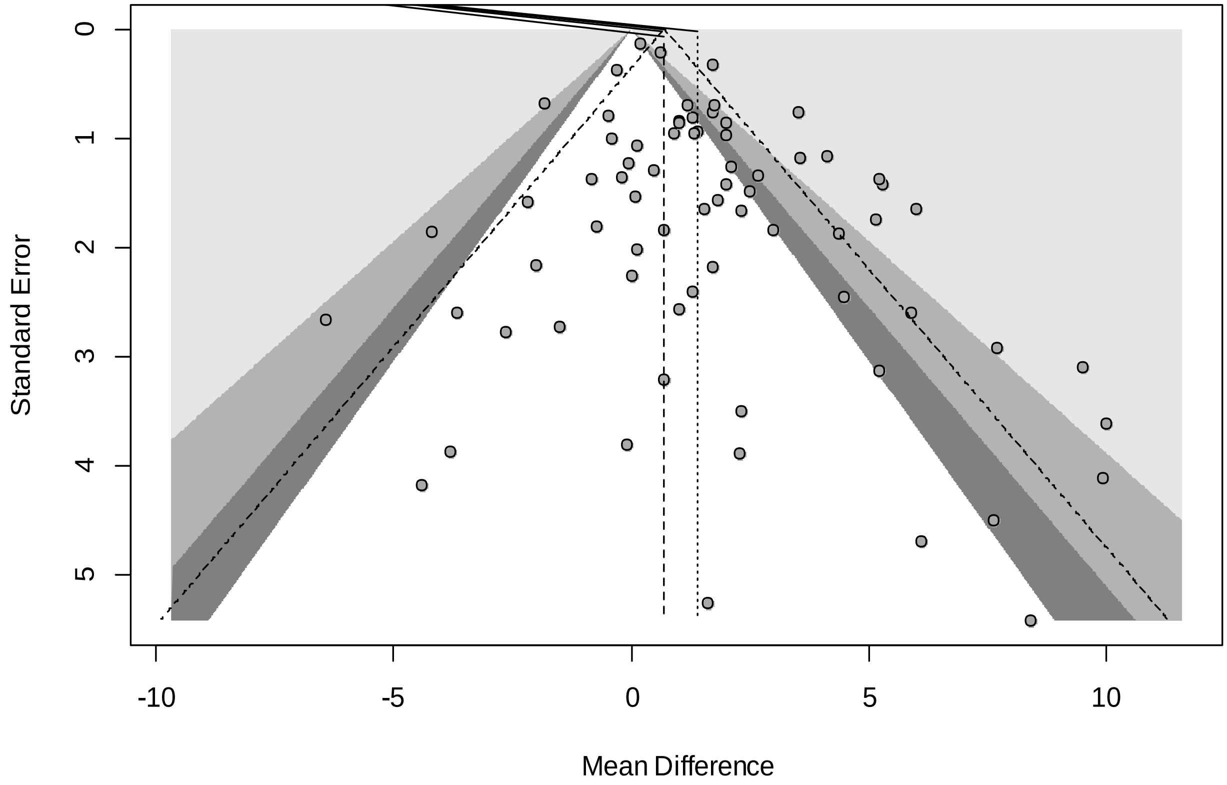

m. Weight

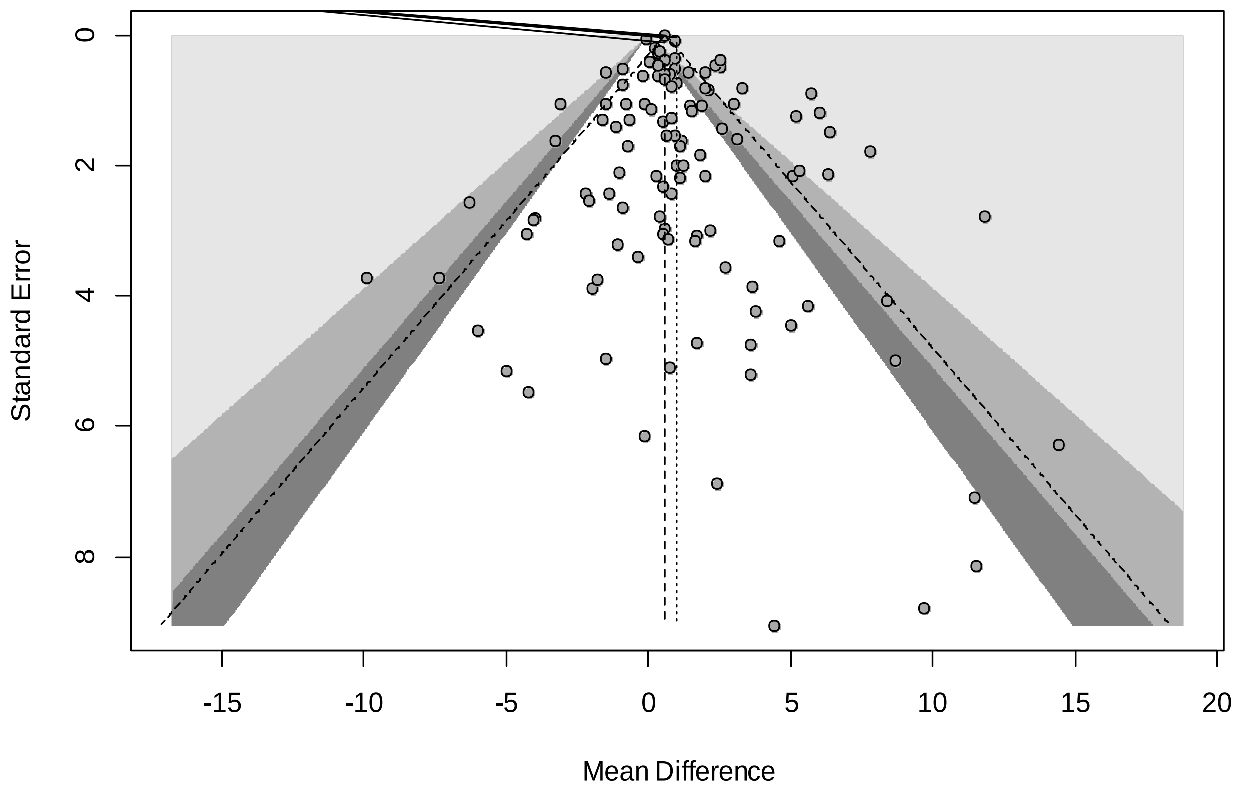

n. Systolic lood pressure

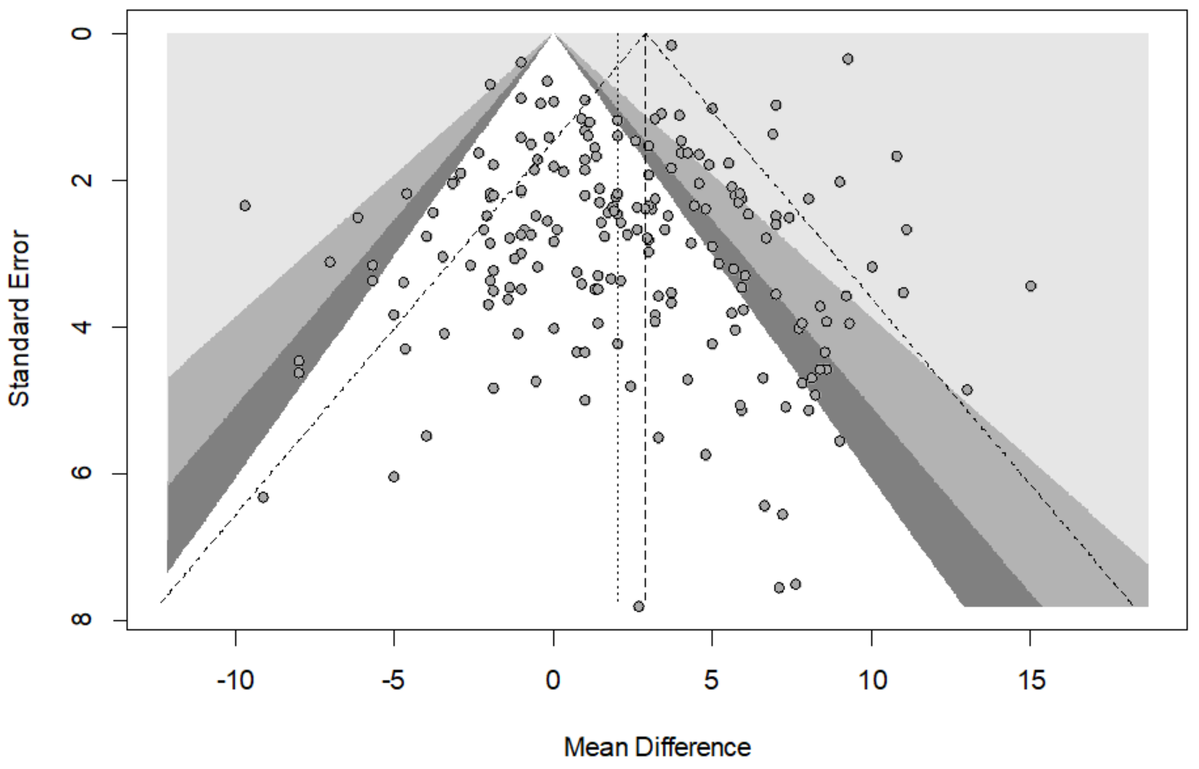

o. **Diastolic blood pressure**

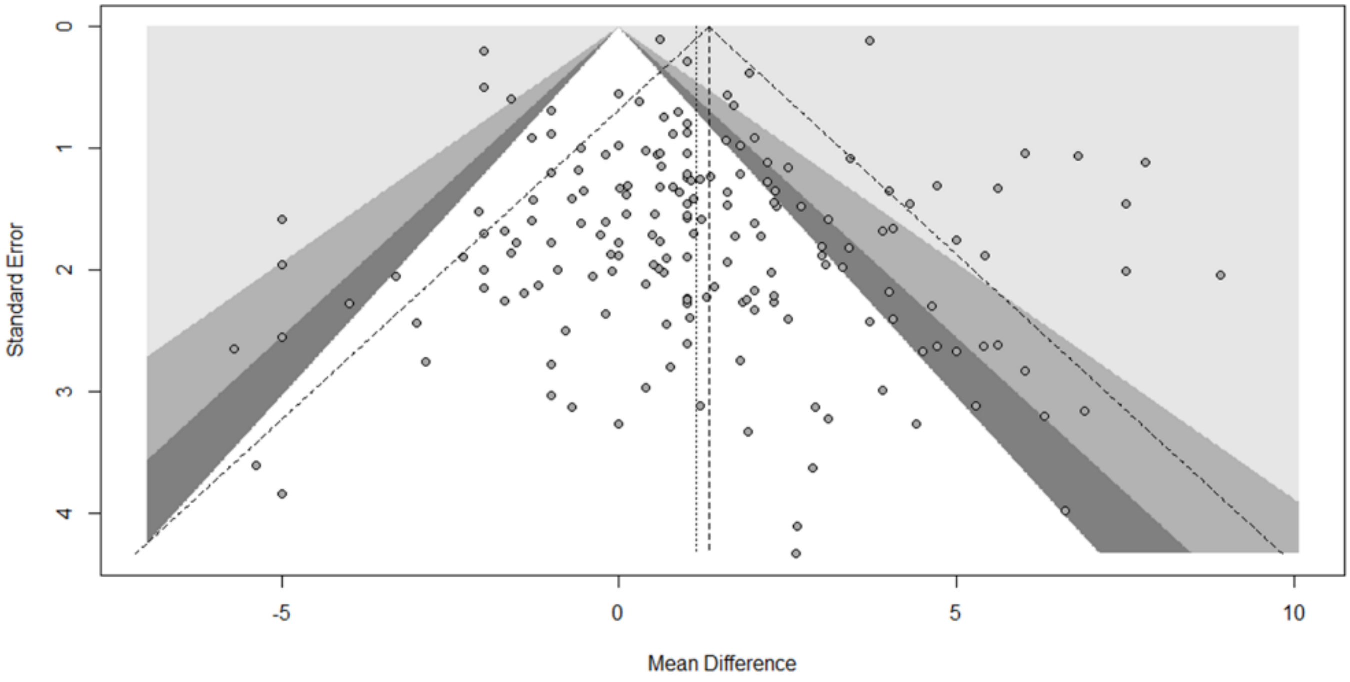

p. **LDL cholesterol**

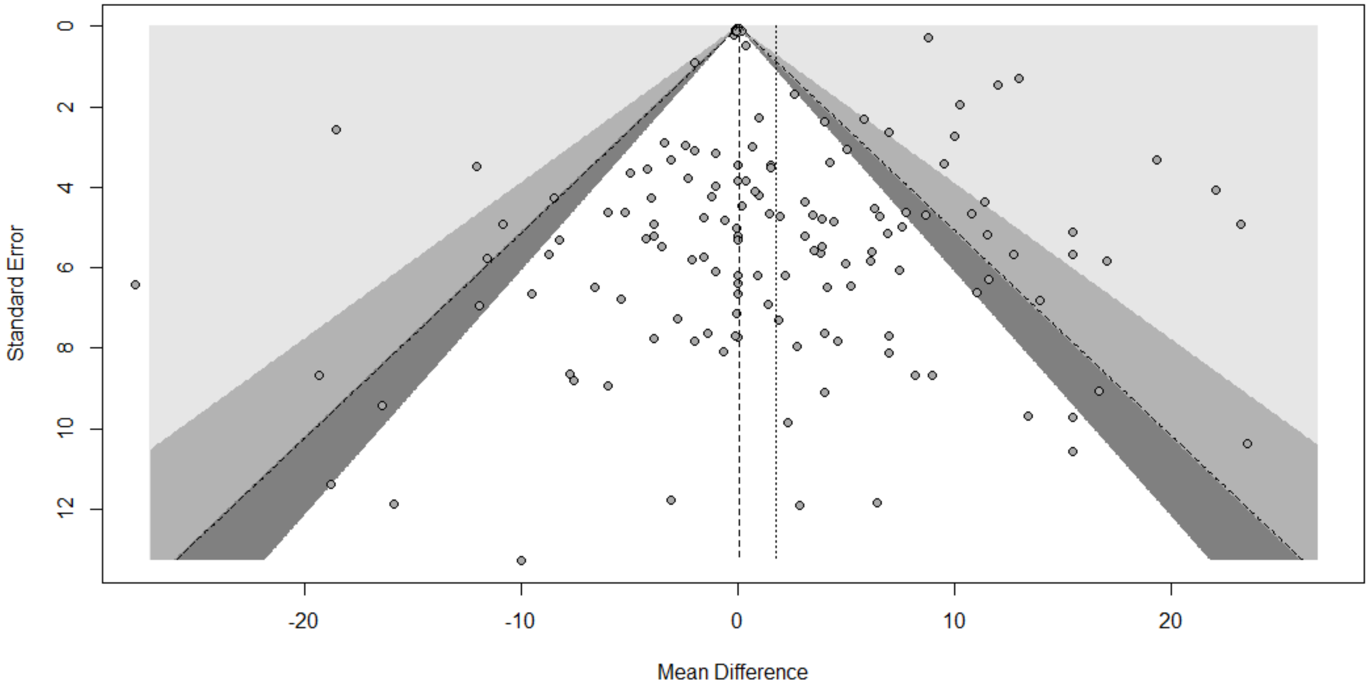

q. HDL cholesterol

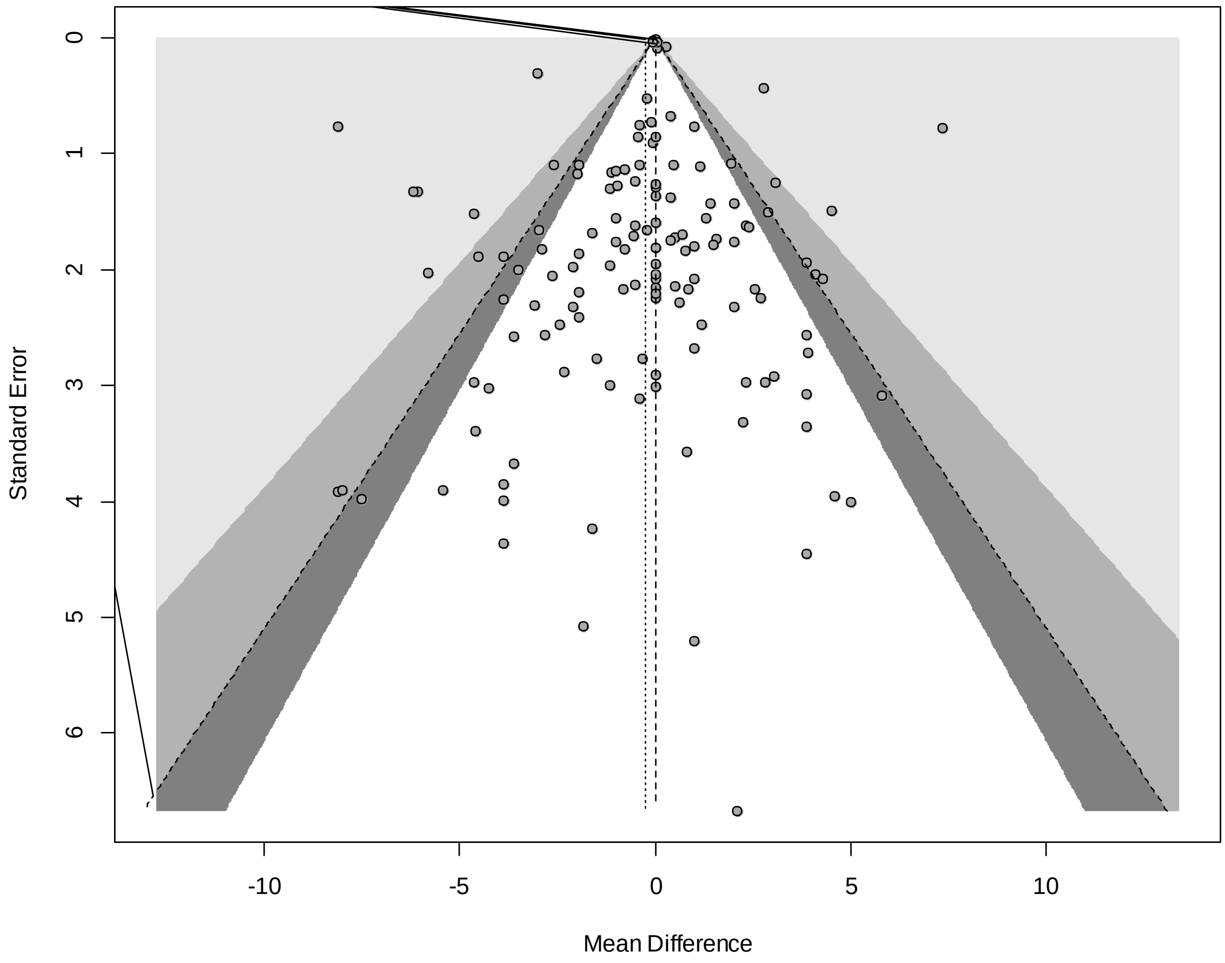

r. Triglycerides

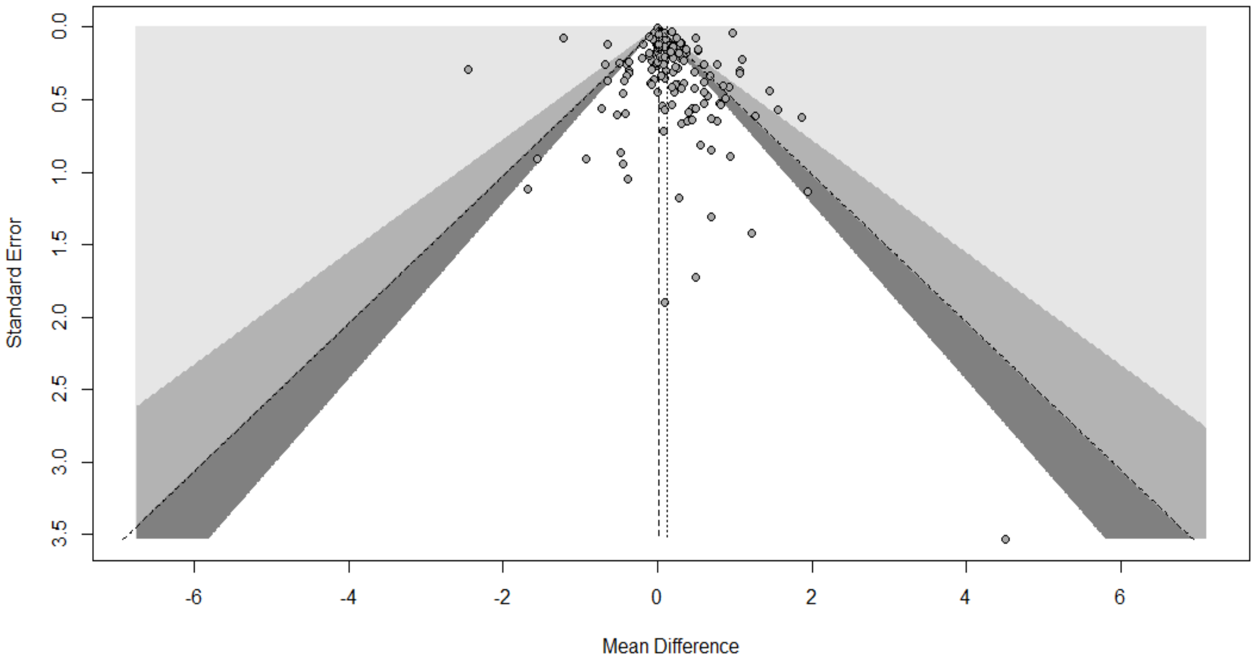

s. Total cholesterol

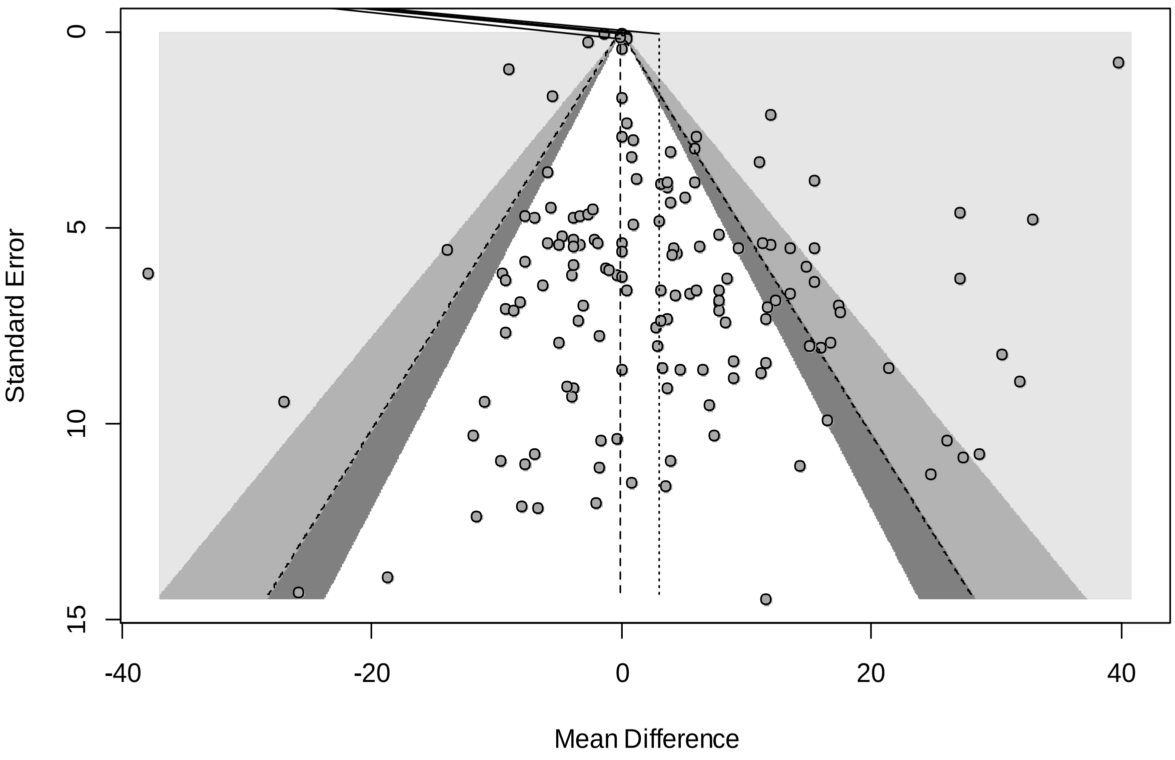

t. Quality of life

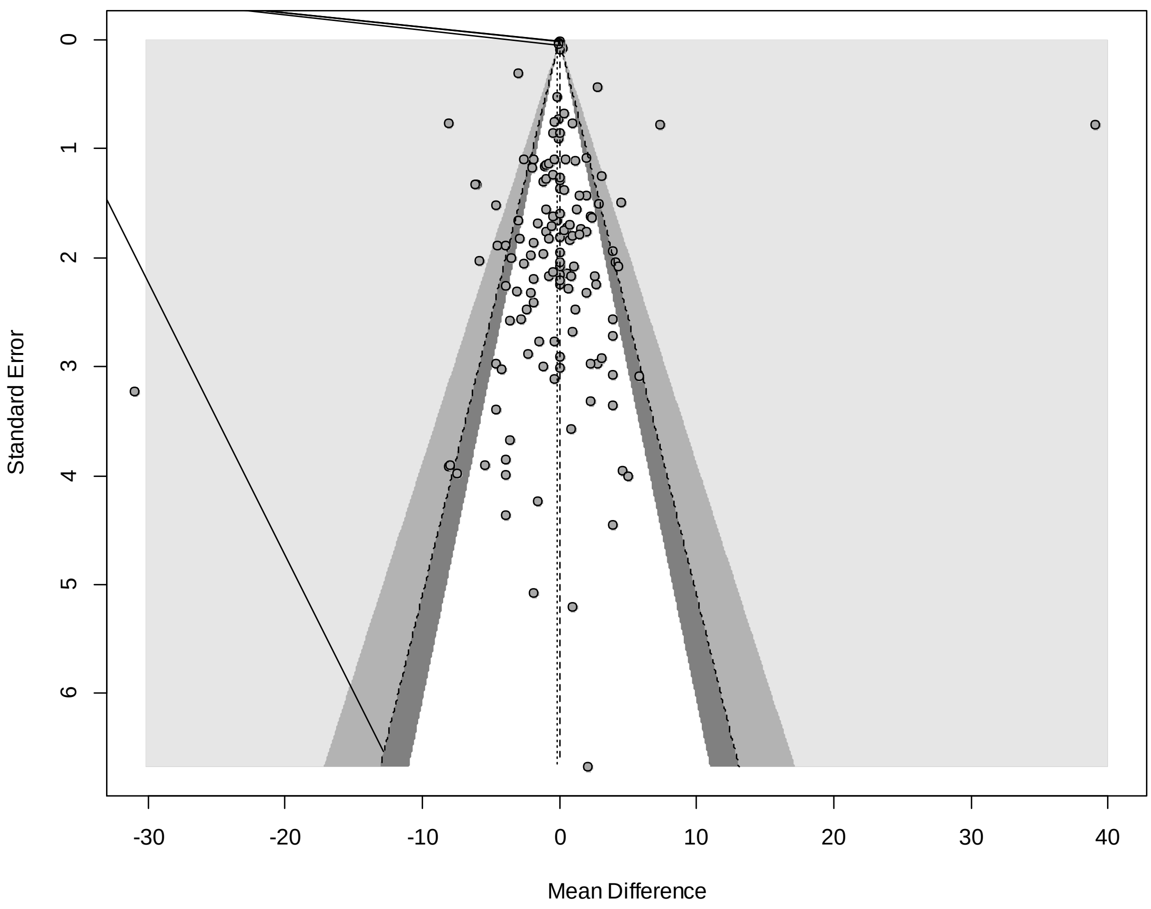

u. Psychological distress

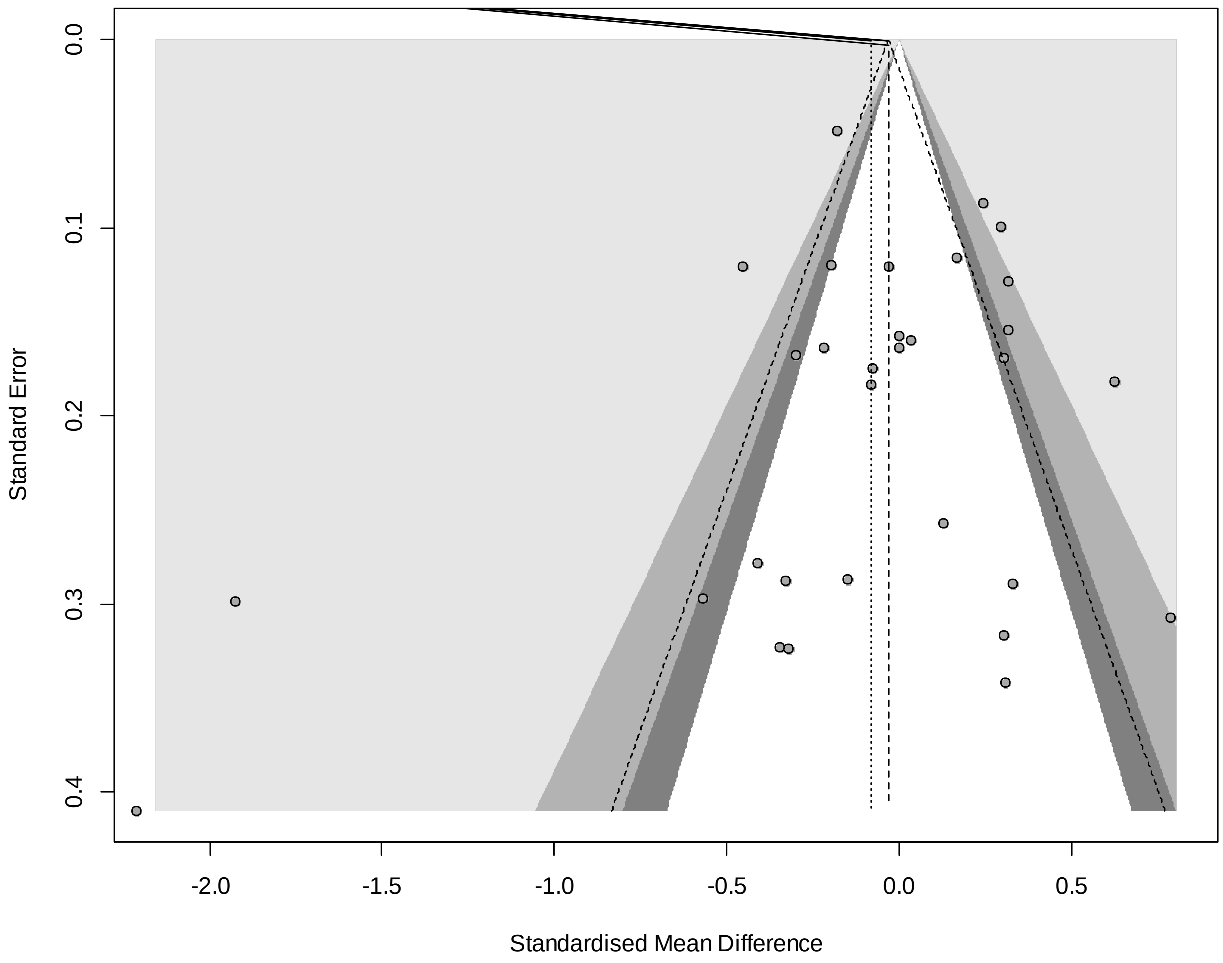

Supplement: Supplementary file 1 [file healthcare-12-00027-s001.zip › Supplementary Materials 18DEC2023.zip!/Supplementary Material S4-Funnel Plots.pdf]
