# Supplementary material for: Exploring the Effectiveness of Self-Management Interventions in Type 2 Diabetes: A Systematic Review and Network Meta-Analysis
Source: Healthcare (Basel). 2023 Dec 22;12(1):27. doi: 10.3390/healthcare12010027 (PMC10779199; doi:10.3390/healthcare12010027)
Supplement: Supplementary file 1 [file healthcare-12-00027-s001.zip › Supplementary Materials 18DEC2023.zip!/Supplementary Material S6-Meta-regression Results.pdf]

Appendix E-Meta-regression results

Meta-regression according to severity

Subcategory	Main Category (outcome)	# studies	TE [95% CI]	τ^2 (I^2)
**Basic empowerment measures**				
Self-management competences	Knowledge	35	0.09 [-0.12, 0.30]	0.43 (92%)
	Self-efficacy	40	0.24 [-0.01, 0.48]	0.41 (94%)
**Adherence to Self-management behaviours**				
	Self-management behaviours	26	-0.03 [-0.19, 0.14]	0.15 (84%)
	Adherence	43	0.13 [-0.10, 0.37]	0.19 (86%)
	Foot care	21	0.10 [-0.54, 0.74]	0.89 (96%)
	Glucose Self-monitoring	25	-0.49 [-1.04, 0.06]	0.63 (95%)
	Dietary habits	24	-0.02 [-0.41, 0.38]	0.32 (89%)
	Consumption of fat	10	-0.08 [-0.20, 0.05]	0.02 (47%)
	Physical activity	44	-0.02 [-0.23, 0.19]	0.26 (88%)
**Clinical outcomes (and markers)**				
Glucose management	Hb1Ac	368	-0.01 [-0.01, 0.00]	0.16 (98%)
Weight management	Body Mass Index (BMI)	192	-0.01 [-0.03, 0.01]	0.53 (93%)
	Waist size	70	0.10 [-0.07, 0.26]	3.10 (81%)
	Weight	106	0.15 [-0.06, 0.36]	3.14 (96%)
Blood Pressure	Systolic blood pressure	188	0.01 [-0.08, 0.09]	8.82 (77%)
	Diastolic blood pressure	170	0.01 [-0.05, 0.07]	3.12 (82%)
Lipid profile	LDL cholesterol	134	-0.09 [-1.24, 1.07]	32.08 (99%)
	HDL cholesterol	131	-0.30 [-0.68, 0.08]	2.97 (99%)
	Triglycerides	133	0.00 [-0.01, 0.01]	0.13 (99%)
	Total cholesterol	146	0.04 [-0.20, 0.27]	79.02 (100%)
**Quality of life**				
	Quality of life	60	0.00 [-0.02, 0.01]	0.30 (92%)
	Psychological distress	27	0.00 [-0.02, 0.01]	0.07 (73%)

TE, Treatment Effect; CI; Confidence Interval

Meta-regression according to gender

Subcategory	Main Category (outcome)	# studies	MD [95% CI]	τ^2 (I^2)
**Basic empowerment measures**				
Self-management competences	Knowledge	8	-0.03[-0.07, 0.02]	0.83 (95%)
	Self-efficacy	16	0.00[-0.01, 0.01]	0.08 (70%)
**Adherence to Self-management behaviours**				

	Self-management behaviours	9	0.00 [-0.02,0.01]	0.15 (87%)
	Adherence	9	-0.01 [-0.02,0.01]	0.21 (56%)
	Foot care	8	-0.01 [-0.08, 0.06]	0.96 (96%)
	Glucose Self-monitoring	5	-0.04 [-0.09, 0.16]	2.58 (98%)
	Dietary habits	6	0.00 [-0.01, 0.01]	000 (0%)
	Consumption of fat	6	0.00 [-0.01, 0.00]	0.00 (13%)
	Physical activity	16	0.00 [-0.02, 0.01]	0.24 (82%)
**Clinical outcomes (and markers)**				
Glucose Management	Hb1Ac	72	0.00 [0.00, 0.01]	0.17 (86%)
Weight management	Body Mass Index (BMI)	40	0.03 [0.01, 0.05]	0.50 (87%)
	Waist size	14	0.08 [-0.05, 0.21]	19.98 (80%)
	Weight	26	-0.03 [-0.05, 0.00]	0.35 (36%)
Blood Pressure	Systolic blood pressure	39	0.06 [-0.02, 0.13]	8.69 (72%)
	Diastolic blood pressure	34	0.05 [0.01, 0.10]	3.44 (71%)
Lipid profile	LDL Cholesterol	25	0.13 [-0.07, 0.33]	18.88 (96%)
	HDL Cholesterol	21	0.05[-0.01, 0.12]	2.52 (95%)
	Triglycerides	20	0.02 [0.00, 0.04]	0.13 (85%)
	Total cholesterol	23	0.08 [-0.15, 0.31]	42.27 (87%)
**Quality of life**				
	Quality of life	13	0.02 [-0.01, 0.04]	0.30 (93%)
	Psychological distress	11	0.01 [-0.01, 0.03]	0.30 (93%)

TE, Treatment Effect; CI; Confidence Interval
